# Modelling water levels of northwestern India in response to improved irrigation use efficiency

**DOI:** 10.1038/s41598-020-70416-0

**Published:** 2020-08-10

**Authors:** Shashank Shekhar, Suman Kumar, A. L. Densmore, W. M. van Dijk, Rajiv Sinha, Manoranjan Kumar, Suneel Kumar Joshi, Shive Prakash Rai, Dewashish Kumar

**Affiliations:** 1 Department of Geology, University of Delhi, New Delhi, Delhi 110007 India; 2 Department of Earth Sciences, Indian Institute of Technology, Kanpur, 208016 India; 3 Institute of Hazard, Risk, and Resilience and Department of Geography, Durham University, Durham, DH1 3LE UK; 4 Water and Environment Division, Arcadis, Piet Mondriaanlaan 26, 3812 GV Amersfoort, The Netherlands; 5 Geological Survey of India, Jaipur, India; 6 Department of Geology, Banaras Hindu University, Varanasi, India; 7 NGRI, Uppal Road, Hyderabad, 500007 India

**Keywords:** Environmental impact, Hydrology

## Abstract

The groundwater crisis in northwestern India is the result of over-exploitation of groundwater resources for irrigation. The Government of India has targeted a 20 percent improvement in irrigation groundwater use efficiency. In this perspective, and using a regional-scale calibrated and validated three-dimensional groundwater flow model, this article provides the first forecasts of water levels in the study area up to the year 2028, both with and without this improvement in use efficiency. Future water levels without any mitigation efforts are anticipated to decline by up to 2.8 m/year in some areas. A simulation with a 20 percent reduction in groundwater abstraction shows spatially varied aquifer responses. Tangible results are visible in a decade, and the water-level decline rates decrease by 36–67 percent in over-exploited areas. Although increasing irrigation use efficiency provides tangible benefits, an integrated approach to agricultural water management practice that incorporates use efficiency along with other measures like water-efficient cropping patterns and rainwater harvesting may yield better results in a shorter period.

## Introduction

**Figure 1:**
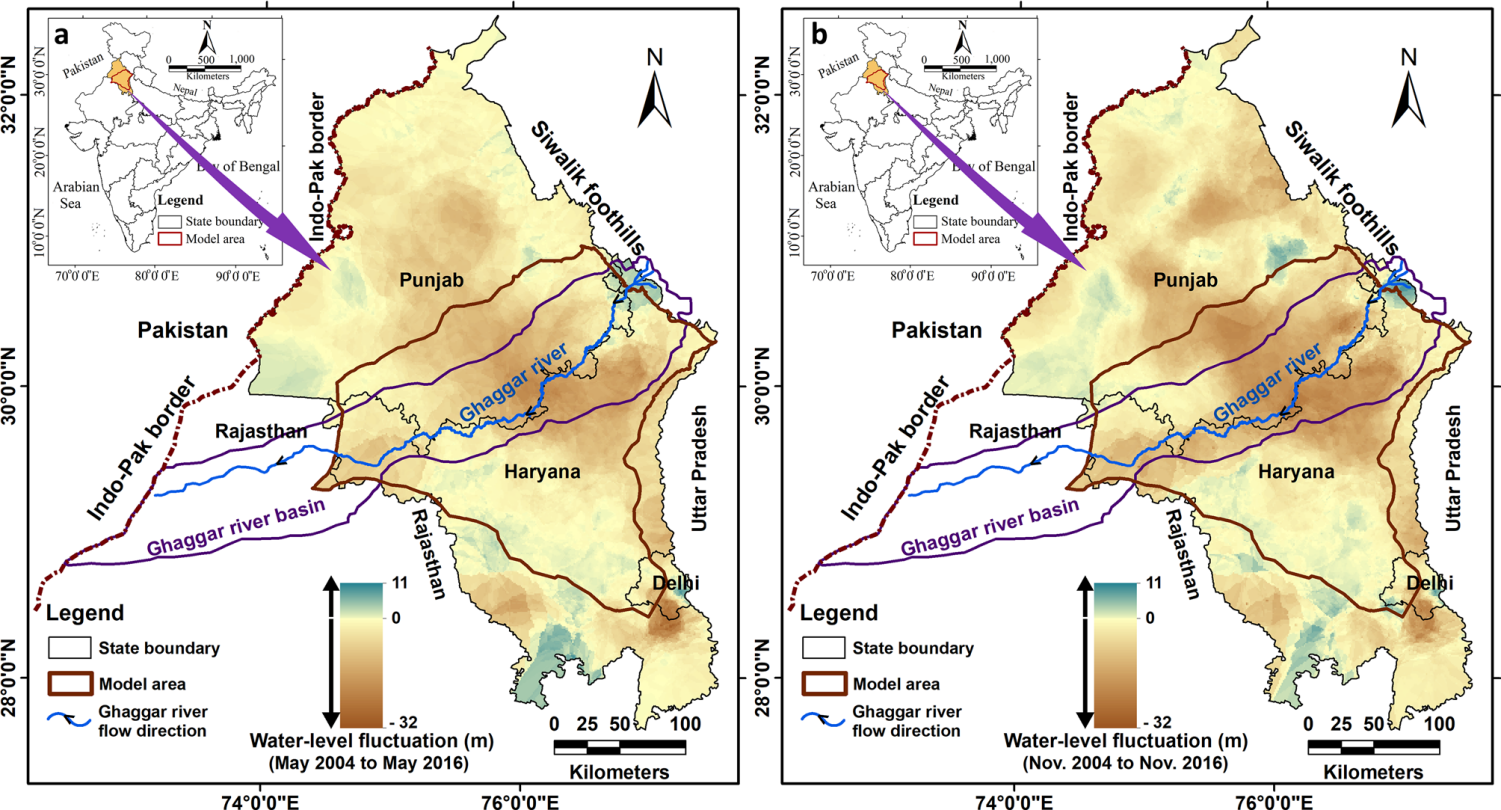
(**a**) Map of Punjab and Haryana states, northwestern India, showing long-term May (pre-monsoon) water-level changes for the period 2004 to 2016. (**b**) Map of Punjab and Haryana showing long-term November (post-monsoon) water-level changes for the period 2004 to 2016. Data available at: https://www.india-ris.nrsc.gov.in/GWLevelApp.html?UType=R2VuZXJhbA==?UName.

The growing world population and food demand have intensified agriculture and irrigation, leading to widespread overexploitation of groundwater resources^1,2^. Northwestern India has emerged as one of the global hotspots of the current groundwater crisis^3–10^. The regional aquifer in northwestern India is a non-indurated alluvial system composed of laterally and vertically heterogeneous deposits of sand, silt and clay, which have been deposited and redistributed by the rivers of the region, with the depositional age of shallow aquifer material varying from recent to 70,000 years^11–13^. The average regional groundwater flow in the study area (Fig. 1) is from northeast (where the saturated aquifer thickness is ~ 300 m) to southwest (saturated aquifer thickness ~ 180 m). In general, the present-day depth to the water table across the region varies in the range of 10–40 m below ground level (mbgl)^8,13^. In a small area in the foothill region in the northeast and in areas with shallow groundwater salinity towards the southwestern fringes of the region, the depth to the water table is in the range of 5–10 mbgl.

Intensive agricultural practice after the Green Revolution in the 1960s is responsible for groundwater depletion in the northwestern Indian states of Punjab and Haryana^13^. The production of wheat in Punjab in 2015–16 (in terms of mass produced per unit area per year) was 49 percent more and rice 65 percent more than the national average of India, while in Haryana wheat and rice production were 42 percent and 28 percent higher, respectively^14^. These high yields are currently sustained by exploitation of groundwater using inefficient flood irrigation methods^15^. Canal irrigation water is unable to meet current irrigation water requirements^16^. Using data from the Central Groundwater Board (CGWB), the nodal agency of the Government of India responsible for groundwater affairs, and state groundwater boards, we estimate that the average canal water released for irrigation in the study area is only about 32 percent of the groundwater abstraction. In addition, the use of canal water for irrigation is highly skewed toward the southern parts of Punjab and Haryana. This increases the dependency of farmers elsewhere in the region on groundwater irrigation. Further, the use of groundwater for irrigation is greatest during the non-monsoon season (November to June with scanty rainfall), particularly for wheat; water requirements for rice during the monsoon season (July to October with plenty of rainfall) are met from a combination of monsoon rainfall and groundwater^4,16^.

**Figure 2:**
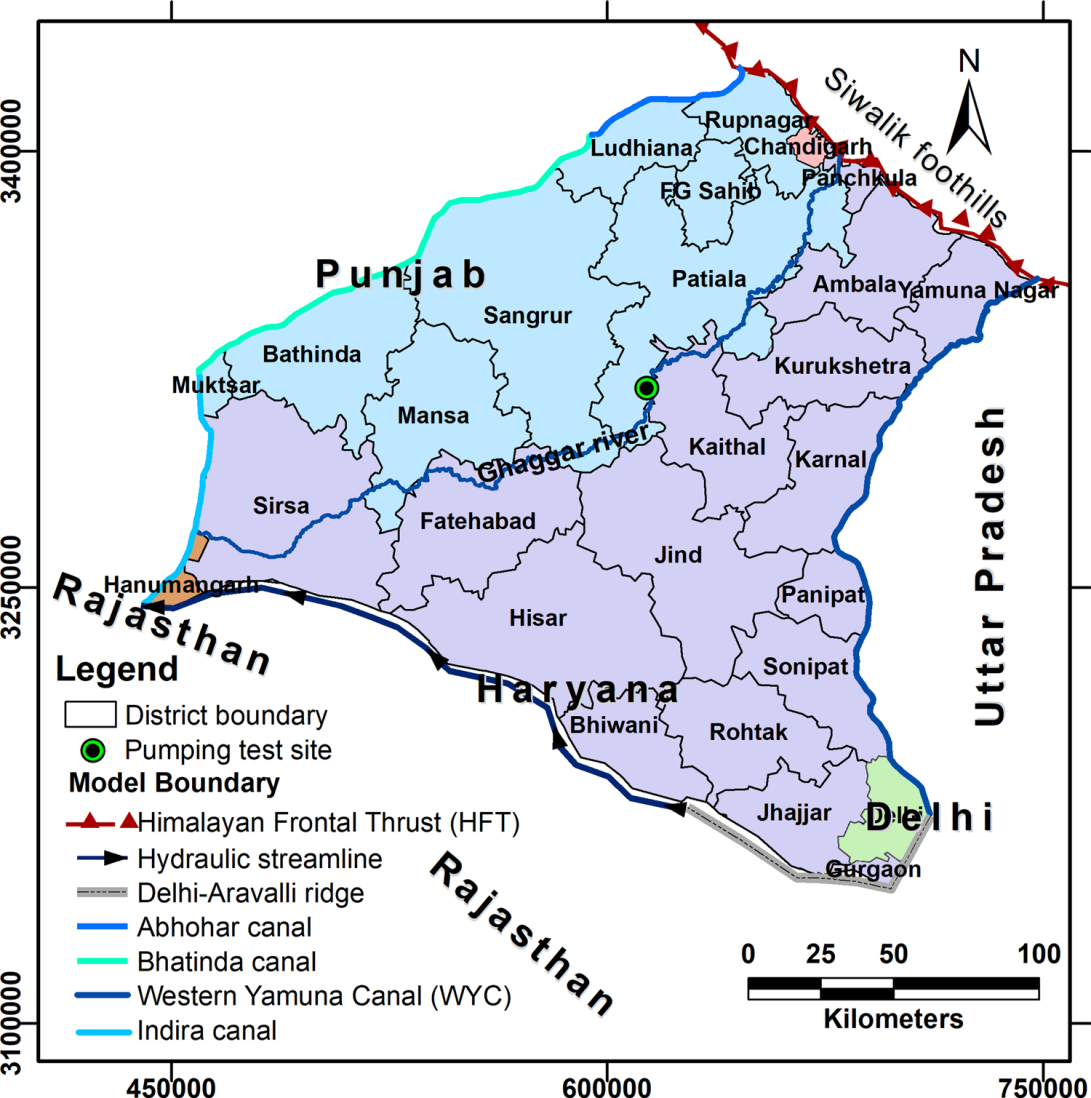
Map showing the model area with district names and model boundaries (Himalayan Frontal Thrust; hydraulic streamline; Delhi Aravalli bedrock ridge; various canals) and the pumping test site in Patiala district.

Groundwater level data show evidence for long-term water-level decline for the years 2004 to 2016 across much of Punjab and Haryana, focused in particular around the districts along and adjacent to the Ghaggar River basin^13^ (Fig. 1). In the majority of these districts (Fatehgarh Sahib, Patiala, Sangrur, Mansa, Sirsa, Ambala, Kurukshetra, Kaithal, and Jind; see Fig. 2) the groundwater level is presently declining (Fig. 1), and the high groundwater abstraction for irrigation in these districts has not stabilised over the years (see Supplementary Table S1). Asoka et al.^16^ and van Dijk et al.^13^ argued that changes in groundwater storage in northwestern India are primarily controlled by pumping rather than by precipitation variation^13,16^. To address this crisis, the Government of India has recommended a 20 percent improvement in irrigation groundwater use efficiency^17,18^. This recommendation is based on recent research which shows that 23–49 percent increases in efficiency can be achieved through alternative irrigation methods (such as sprinkler, drip, alternate wetting/drying approaches), with no loss in yield^19,20^. The impact of this proposed improvement on regional-scale water levels, however, has not yet been quantitatively assessed. Thus, in this paper, we seek to explore the potential benefits of the proposed improvement. We address two questions: 1. Will improving irrigation efficiency decrease the rate of water-level decline, or has groundwater exploitation crossed a threshold of aquifer resilience? 2. How will the aquifer response to improved irrigation efficiency vary across different parts of the study area? To answer these questions, we modelled the regional groundwater system with the following main objectives:

1. Determine how groundwater levels will change in the next decade (2020–2030) at the present rate of groundwater abstraction; and
2. Assess the spatial and temporal impacts of the proposed improvement in agricultural groundwater use efficiency by 20 percent over the same period.

The 20 percent improvement in the agricultural groundwater use efficiency was modelled as a 20 percent reduction in the groundwater abstraction for irrigation use. While there have been some prior efforts to look at water-level variations in northwestern India, there have been no efforts to model three-dimensional groundwater flow on a regional scale, nor have there been a quantitative evaluations of any proposed mitigation strategy. This paper reports one of the first solution-oriented research studies for India using a calibrated and validated (for the years 2004 to 2017), regional-scale three-dimensional groundwater flow model, similar to recent studies in the North China plain^1^ and the high plains of the USA^2^.

## Methods

The regional groundwater flow model covers an area of 44,192 km^2^ and includes large parts of Punjab and Haryana and small portions of Rajasthan and Delhi. The regional model is enclosed between the Himalayan Frontal Thrust to the northeast, the Western Yamuna Canal to the east, the Abhohar and Bhatinda Canal to the northwest, and the Indira Gandhi Canal to the west (Fig. 2). These canals were chosen as the model boundaries, as they have voluminous water flows throughout the year and significant seepage of water into the groundwater system, leading to recharge of the aquifer. We did an extensive field survey to collect data on water stage, wetted perimeter and cross-sectional area of the canals. These canal data were incorporated into the model to set the boundary condition.

Furthermore, based on the regional groundwater flow assessed from water-level contour maps for the years 2004, 2009, and 2011 (see Supplementary Figs. S1, S2 and S3), a no-flow hydrological boundary (shown as a hydraulic streamline in Fig. 2) was assigned to the southwestern side of the model. The model grid cells along this boundary were assigned variable heads in different stress periods during the simulation exercise. The southeastern model boundary was similarly set as a no-flow boundary along the Delhi-Aravalli bedrock ridge. The Ghaggar River is a seasonal river that flows through the centre of the model space, and is dry during summer in many stretches; no seasonal discharge data are currently available. The groundwater levels in the Ghaggar basin have declined significantly, and the river is mostly disconnected from the groundwater system^21^. Hence, the river was not incorporated as a boundary in the groundwater model. Instead, recharge from the river was simulated by incorporating approximate periodic seasonal recharge into grid cells along the river course.

In order to develop the regional groundwater model, we had to determine the extent to which the aquifer system can be described as a laterally-continuous regional confined or unconfined aquifer, or as an open, heterogeneous anisotropic system. Earlier studies^11^ in the study area have characterised the aquifer geometry in the top 200 m as stacked sand bodies with variable vertical connectivity. It has also been shown that deeper groundwater in northwestern India, up to a depth of 320 mbgl, has a significant component of recent recharge by vertical leakage^22,23^. Based on these findings, we assumed that the regional system consists of a heterogeneous anisotropic aquifer in the form of stacked, vertically connected aquifer bodies. In order to confirm the vertical connectivity of the aquifer by observing the water-level response, we performed an in situ experimental investigation in the field.

## Experimental investigation

**Figure 3:**
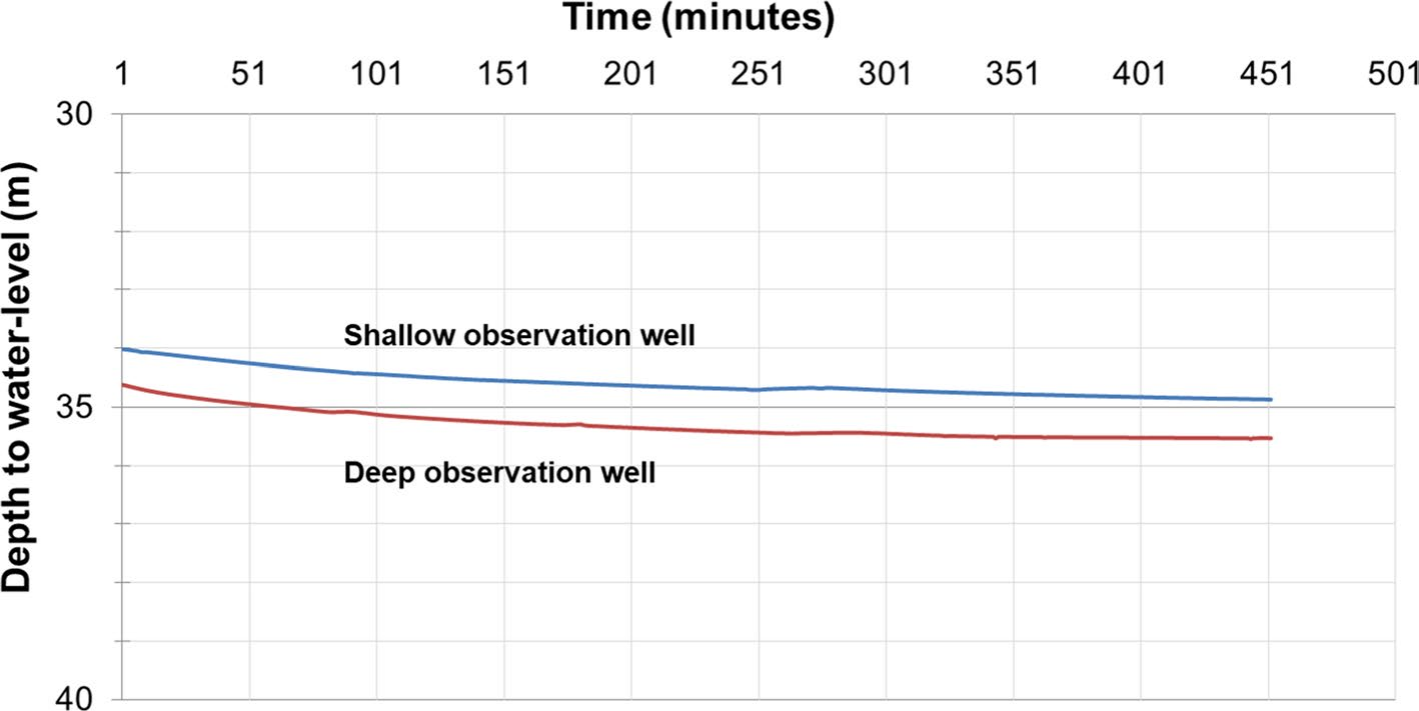
A plot of depth to water-level with respect to time (minutes) in shallow (depth range of 40–44 mbgl, blue line), and deep (depth range of 87–91 mbgl, red line) observation wells after the commencement of pumping at Seona village, Badshahpur, Patiala district, Punjab.

We carried out a pumping test at a site in Seona village, Badshahpur, Patiala district, Punjab; roughly in the centre of the study area (Fig. 2). The pumping well (PW) was designed to extract groundwater from a depth range of 76–86 mbgl, and two observation wells (OW) were placed at depth ranges of 40–44 and 87–91 mbgl, respectively (Supplementary Fig. S4; Table S2). The pumping well was continuously pumped at a discharge of 288 m^3^/day for 452 min. The changes in the groundwater levels for both shallow and deep observation wells were continuously recorded at regular intervals and are shown in Fig. 3.

We observed that, in response to pumping, the water levels in both shallow and deep observation wells had similar rhythmic fluctuations (Fig. 3). This rhythmic fluctuation confirms hydrogeological connectivity between depths of 40 and 91 m at the pumping test site. This result is consistent with the inference drawn based on lithological data from 240 wells distributed over the study area, for depths of up to 200 m, that the study area is underlain by a single heterogeneous and anisotropic aquifer^11^. The vertical connectivity of the aquifer system was also confirmed using isotopic data from 16 paired locations^22^. Moreover, isotopic analysis of samples from 244 locations in the Ghaggar basin (occupying the central part of our study area; see Fig. 1) indicates that modern meteoric water is a significant source of groundwater recharge for the shallow and deep aquifers, showing that regionally the aquifer is unconfined^23^. For the regional groundwater model, we, therefore, assumed one aquifer which is regionally unconfined, heterogeneous, and anisotropic.

## Groundwater modelling

Simulating a regional aquifer system where heterogeneity and anisotropy are defined by elongate sand bodies in a framework of fine sand and silt together with clay lenses is computationally challenging^11^. It ideally would require an extensive database of lithologs to produce reasonable three-dimensional extrapolation for defining the correct aquifer geometry. For example, if a clay layer of 2 m thickness is noticed in two different lithologs from boreholes 8 km apart, this could be extrapolated as a continuous clay sheet, while in reality, it could be two narrow, elongated small clay bodies separated by silty or sandy formations. Consideration of this uncertainty is important; on the one hand, interpolation of a regional clay body could lead to the interpretation of a regionally-significant confining layer, while in reality, the aquifer may be regionally unconfined with clay lenses. Given the limited availability of both lithologs and accompanying aquifer parameter data in this relatively extensive regional model area, any three-dimensional extrapolation may produce a wrong replica of the real aquifer geometry, leading to a complex descriptive model of the groundwater system with false spatial pattern and wrong parameter values^24^. In this context, with the present constraints in the extrapolation of the regional aquifer geometry^25^, it would be more sensible to simulate aquifer geometry for the regional groundwater flow model as a proxy homogenous, and anisotropic unconfined aquifer with predominantly vertical anisotropy. To assign this vertical anisotropy to the model grid cells, we used published results^26^ for alluvial deposits in the neighbouring area north of Delhi, where vertical hydraulic conductivity was determined to be approximately one-tenth of the horizontal hydraulic conductivity. This pattern is reasonable, given that the regional aquifer system consists of stacked, unconsolidated to weakly-consolidated fluvial sediments.

The use of a more simplistic representation of the aquifers is justified as, during the process of model calibration, the aquifer parameters are further changed. A calibrated groundwater flow model is at best an approximation of the real system where the system response to changes in input and output stresses is near to reality. Calibrated aquifer parameters reflect one average value with vertical anisotropy for the whole aquifer system, which is similar to the approach used for large scale groundwater flow model in the neighbouring Delhi region^26^. This approach allows us to get indicative answers to the questions and objectives defined above.

GW Vistas was used for the regional groundwater flow modelling. MODFLOW, which simulates three-dimensional groundwater flows by discretising the flow equation in space, and time using a finite difference method, was used to simulate groundwater flow^27^. For simulation of the groundwater system, MODFLOW solves the general partial differential equation for a constant density three-dimensional groundwater flow in space and time (Eq. 1):

1 $$\frac{\partial }{\partial x}\left({K}_{\mathit{xx}}\frac{\partial h}{\partial x}\right)+\frac{\partial }{\partial y}\left({K}_{\mathit{yy}}\frac{\partial h}{\partial y}\right)+\frac{\partial }{\partial z}\left({K}_{zz}\frac{\partial h}{\partial z}\right)+W={S}_{s}\frac{\partial h}{\partial t}$$

where K_xx_, K_yy_, and K_zz_ are values of hydraulic conductivity along the x, y, and z coordinate axes, which are assumed to be parallel to the principal axes of hydraulic conductivity (L T^−1^); h is the water level head (L); W is a volumetric flux representing sources and/or sinks of water (T^−1^); S_s_ is the specific storage of the porous material (L^−1^); and t is time (T).

Groundwater use and groundwater recharge data were available for the year 2004 onwards (Supplementary Tables S1 and S3), and water-level data were also available for the period (Supplementary Table S4). Thus, we set 1 July 2004 as the baseline datum and proceeded with the steady-state simulation of groundwater flow.

### Steady-state model

The objective of steady-state modelling was to generate initial water-level head values in the model grid cells for transient modelling. All of the input and output stresses of the groundwater system were incorporated in the single-layer model for one year starting 1 July 2004. District-wise groundwater abstraction and recharge data from the report of the CGWB^28^ were used to set up the steady-state model (Supplementary Tables S1 and S3).

**Figure 4:**
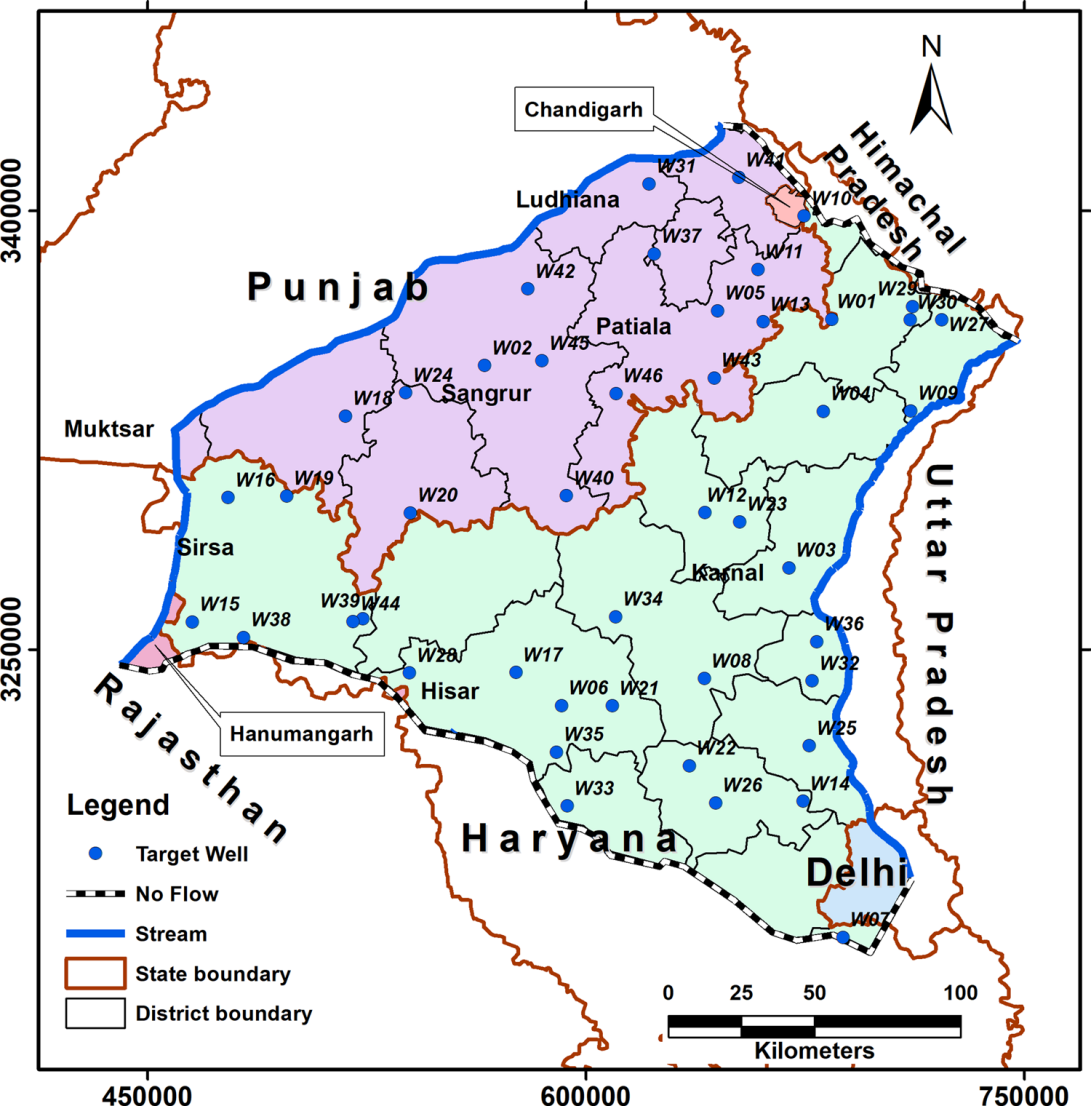
Map showing the model boundaries and the location of the 46 CGWB target/observation wells used in the model calibration and validation.

Since, negligible groundwater flow is expected across the Himalayan frontal thrust in the northeast of the study area, the hydraulic streamline, and the Delhi Aravalli ridge in the southwest (Fig. 2), we simulated these as no-flow boundaries (Fig. 4).

For a realistic simulation, the canals were simulated as streams rather than as constant head boundaries (Fig. 4). The model grid cells were set to a horizontal resolution of 6 × 6 km. We incorporated 46 representative groundwater level monitoring stations run by the CGWB, and that have water-level data for a sufficiently long time over the simulation period, as target wells for calibration of the model (Fig. 4). Elevations of the model top surface were extracted from Shuttle Radar Topography Mission digital elevation model data. As there are insufficient subsurface data to define the bottom of the aquifer system, the base of the model was set to mean sea level. After incorporation of the aquifer parameters (Supplementary Table S5), the steady-state model generated head for August 2004 was calibrated with the help of observed water-level heads from the 46 CGWB stations for that period (Supplementary Figs. S5 and S6). The calibration yielded a determination coefficient of 0.99 between modelled and observed head values (Supplementary Fig. S6).

### The transient groundwater flow model

The single layer steady-state model was adapted into a transient model in GW Vistas. We imported the initial head from the calibrated steady-state model, and used district-wise recharge and groundwater abstraction data from CGWB reports (Supplementary Tables S1 and S3). The CGWB reports have the advantage that they have one recharge value which includes recharge from precipitation, recharge from canal seepage, and direct normative estimates of recharge from irrigation return flow. To the best of our knowledge, this is the first regional groundwater flow model in India that accounts for irrigation return flow in groundwater recharge. Initially, the boundary of each district was digitised as a zone or reach for assigning recharge and groundwater abstraction values in the model (Fig. 2). While the CGWB reports give an average value for the whole district, we noticed variations in groundwater abstraction within districts during our field survey. Thus, during the process of calibration, the zones of groundwater abstraction coinciding with district boundaries were either slightly extended beyond the boundary or reduced. It is also important to note that the CGWB data segregate groundwater recharge and abstraction values for the monsoon and non-monsoon seasons^28–31^ (Supplementary Tables S1 and S3). However, these are time-averaged values and do not reflect the actual temporal variations in abstraction or recharge during these seasons. Besides, our field survey revealed that groundwater abstraction and recharge are not constant throughout the monsoon and non-monsoon seasons. As an approximation, we used agricultural practices and rainfall patterns of the study area to divide each climatic year (July to June) into six stress periods (Table 1). We assigned monsoon and non-monsoon recharge and abstraction values to these stress periods on a pro-rata basis, using the prevalent agricultural practices during each period (Table 1).

**Table 1:** Stress periods and the norms for segregation of groundwater recharge and abstraction from the lumped CGWB district-wise estimates.

Stress period	Month	Groundwater abstraction assigned	Groundwater recharge assigned
1	July–August	Total monsoon groundwater abstraction	Half of the monsoon recharge
2	September–October	Zero groundwater abstraction during the crop harvesting period	Half of the monsoon recharge (before crop harvesting)
3	November–December	Half of the total non-monsoon groundwater abstraction	One-third of the non-monsoon recharge
4	January–February	Half of the total non-monsoon groundwater abstraction	One-third of the non-monsoon recharge
5	March–April	Zero groundwater abstraction during the crop harvesting period	One-third of the non-monsoon recharge
6	May–June	Zero groundwater abstraction	No recharge

Initially, for calibration, the transient model was set up for the period July 2004 to June 2009. Then the model was extended and validated up to August 2017. Calibration and validation were based on 1786 available water-level measurements for January, May, August, and November for the period 2004 to 2017 from 46 target wells of CGWB (see Fig. 4 for their location and Supplementary Table S4).

District-wise groundwater recharge and abstraction data were available only for the years 2004, 2009, 2011, and 2013. Because the study area is mostly under agricultural activity, we estimated that the average groundwater abstraction for domestic use in the model area is only 5 percent of the abstraction for agricultural use. So for the regional model at the initial stage of data entry, only agricultural groundwater abstraction was considered. However, during calibration of the water level in a few prominent towns, the initial groundwater abstraction value was increased by the addition of a term representing local groundwater abstraction for domestic uses. For the transient groundwater flow model, we needed recharge and abstraction data for all six stress periods defined in Table 1 for the duration July 2004 to August 2017. Therefore, we extrapolated the district-wise groundwater recharge and abstraction data available for the years 2004, 2009, 2011, and 2013 to span the entire model run. To determine the best extrapolation method, we examined the spatio-temporal variation in abstraction by estimating the unit groundwater abstraction (m^3^/km^2^/day) for monsoon and non-monsoon seasons using Eqs. (2) and (3):

2 $$Unit \,groundwater \,abstraction \,for \,monsoon=\left(\frac{Total \,monsoon \,agricultural \,abstraction}{Area \,under \,cultivation\times 120 \,days}\right)$$

3 $$Unit\, groundwater \,abstraction \,for \,non{\text{-}}monsoon=\left(\frac{Total \,non{\text{-}}monsoon \,agricultural \,abstraction}{Area \,under \,cultivation\times 180 \,days}\right)$$

**Figure 5:**
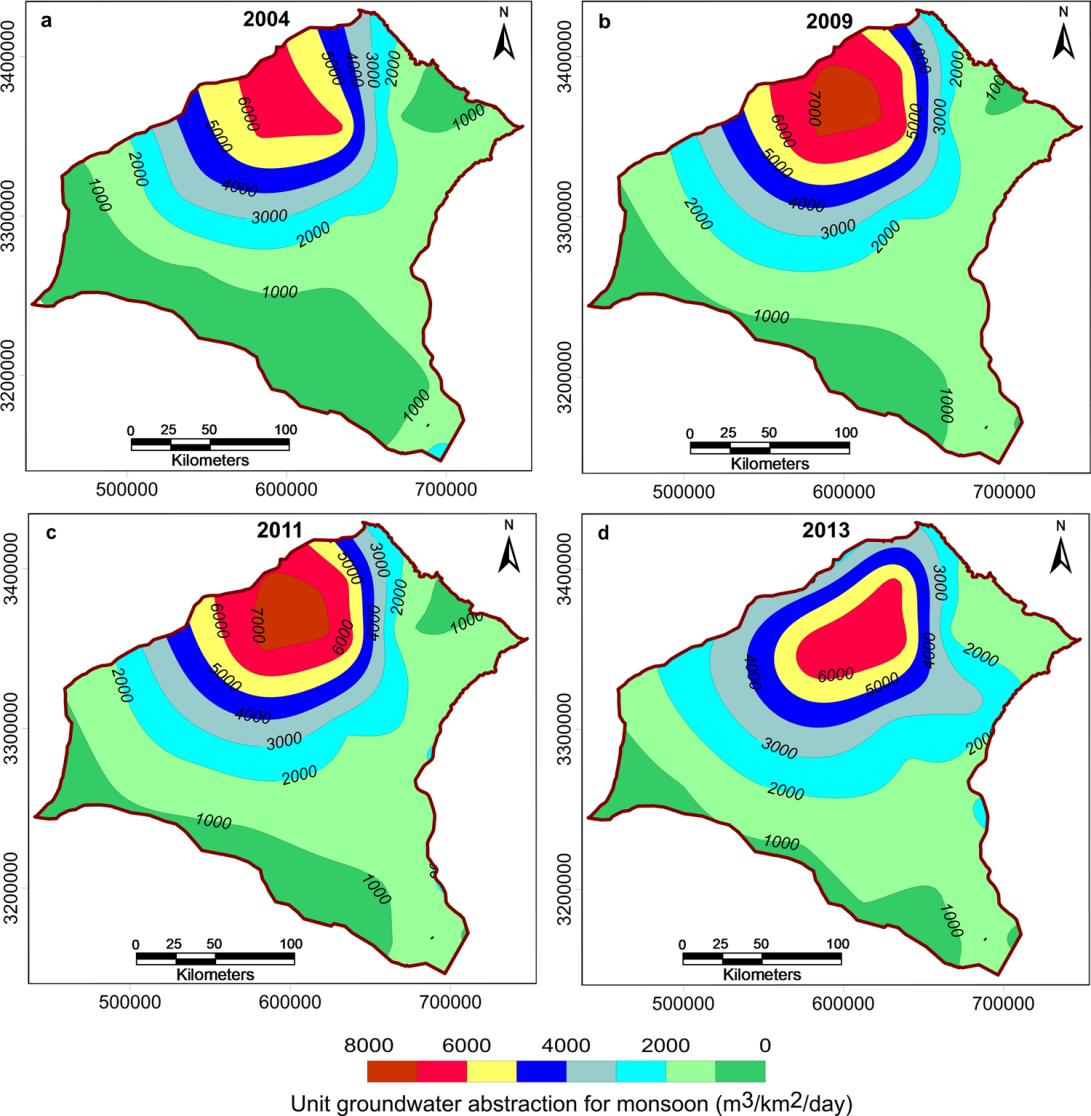
Map showing spatio-temporal variation in unit groundwater abstraction (m^3^/km^2^/day) for the monsoon season in the study area for the years 2004, 2009, 2011, and 2013.

**Figure 6:**
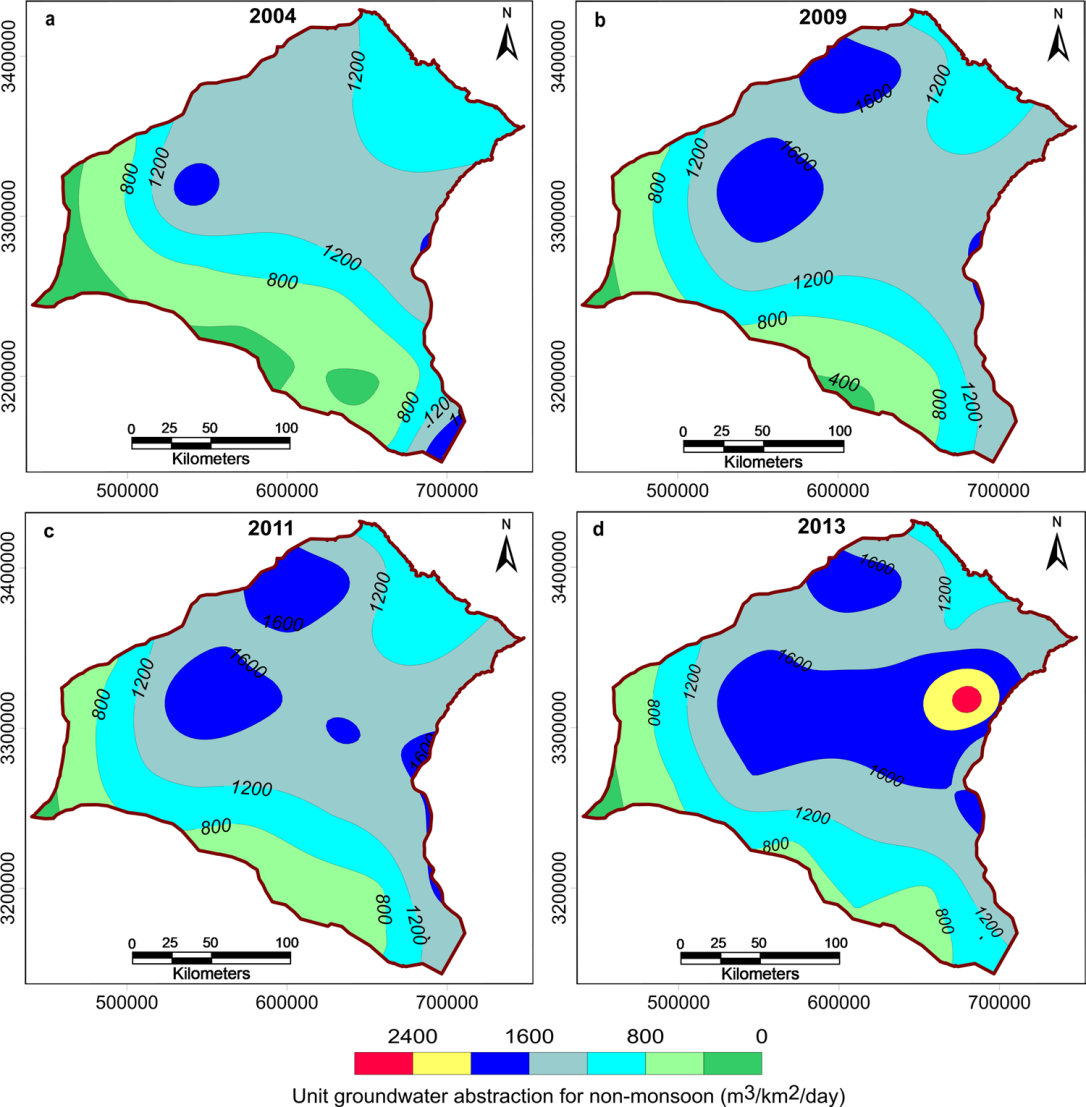
Map showing spatio-temporal variation in unit groundwater abstraction (m^3^/km^2^/day) for the non-monsoon season in the study area for the years 2004, 2009, 2011, and 2013.

We estimated unit groundwater abstraction for monsoon and non-monsoon seasons for each district of the study area. Then in Surfer-10, each district headquarters was assigned the values corresponding to the district, and we determined spatio-temporal variations in the unit groundwater abstraction for monsoon and non-monsoon seasons across the study area for 2004, 2009, 2011, and 2013 using a continuous kriging approach (Figs. 5 and 6). A closer look at the maps in Figs. 5 and 6 reveals that the pattern of groundwater abstraction did not change much from the year 2009 to 2011, although there was some variation before and after this time interval. So for the period July 2004 to June 2009, the groundwater abstraction corresponding to the year 2004 was incorporated in the model, while for the period July 2009 to June 2013, the groundwater abstraction corresponding to the year 2011 was used. From July 2013 to August 2017, values corresponding to the year 2013 were used.

The same approach was used to extrapolate the district-wise CGWB groundwater recharge data available for the years 2004, 2009, 2011, and 2013. We examined the spatio-temporal variation in unit recharge (m^3^/km^2^/day) for monsoon and non-monsoon seasons, estimated using Eqs. (4) and (5):

4 $$Unit \,monsoon \,recharge =\left(\frac{Total \,monsoon \,recharge}{Geographical \,area \,of \,district\times 120 \,days}\right)$$

5 $$Unit\, non{\text{-}}monsoon \,recharge =\left(\frac{Total \,non \,monsoon \,recharge}{Geographical \,area \,of \,district\times 180 \,days}\right)$$

**Figure 7:**
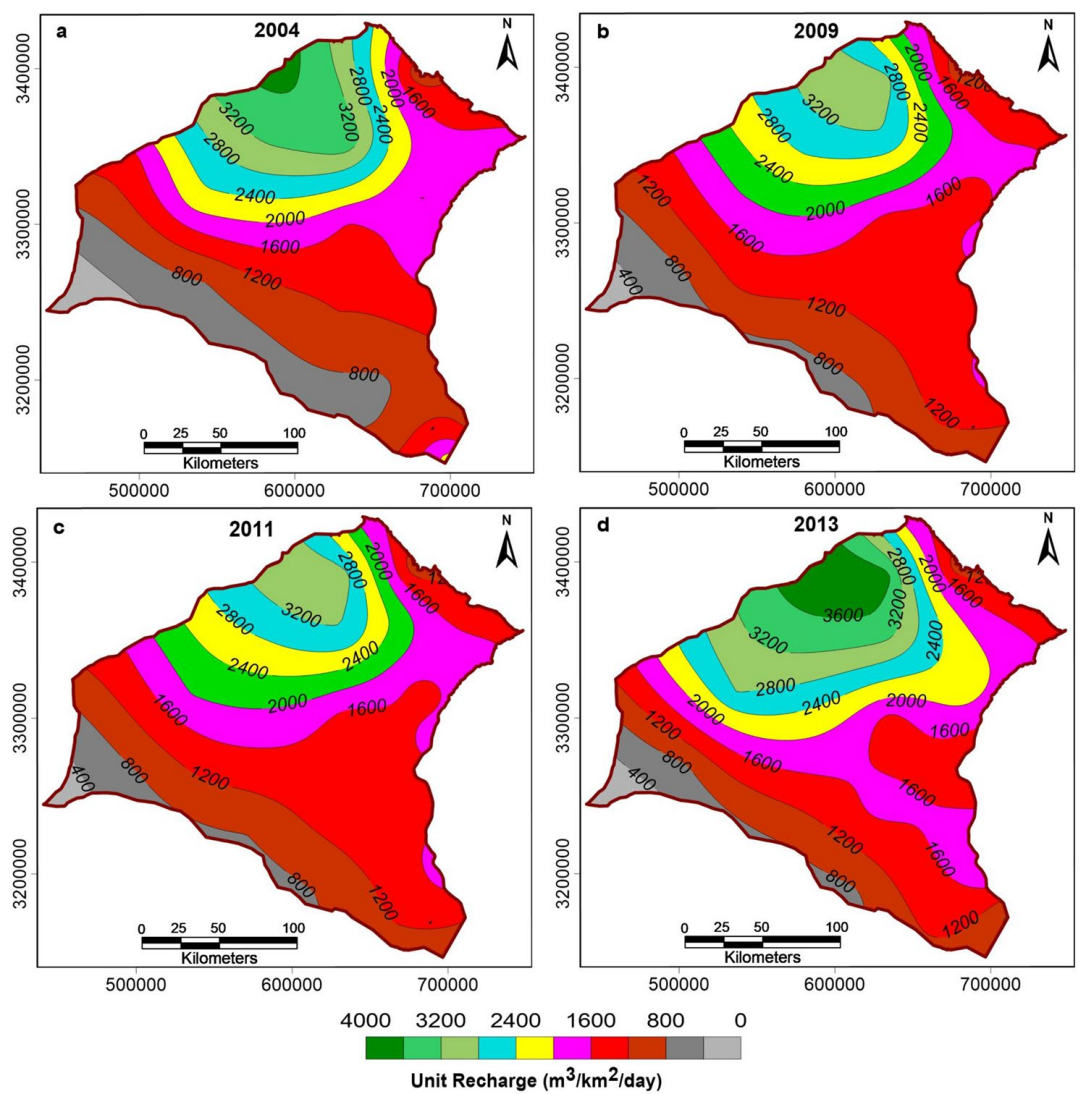
Map showing spatio-temporal variation in unit monsoon recharge (m^3^/km^2^/day) in the study area for the years 2004, 2009, 2011, and 2013.

**Figure 8:**
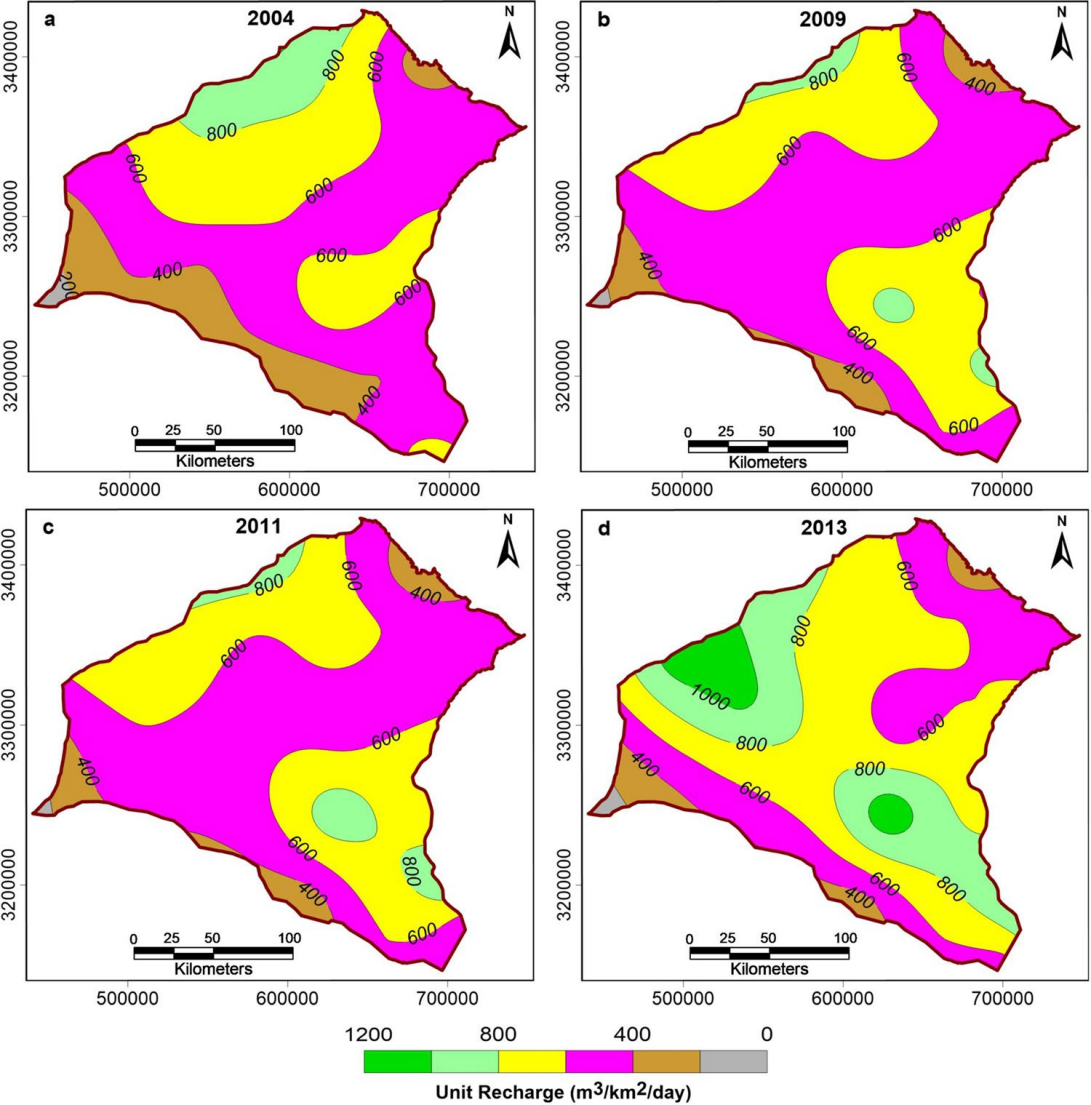
Map showing spatio-temporal variation in unit non-monsoon recharge in the study area for the years 2004, 2009, 2011, and 2013.

We generated maps showing spatio-temporal variations in unit monsoon and non-monsoon recharge for the study area for the years 2004, 2009, 2011, and 2013 (Figs. 7 and 8). Groundwater recharge did not show any significant change for the years 2004, 2009, 2011, and only minor changes in the year 2013. The small changes in the year 2013 were ignored as they are unlikely to make a significant impact on the regional scale. Thus, the recharge figures estimated for the year 2004 were incorporated in the model for the whole duration over 2004 to 2028. This choice is also justified by estimates that the changes in the groundwater storage in northwestern India are primarily controlled by pumping rather than by precipitation variation^16^. As this study is an initial exploration of the impacts of future groundwater management strategies, we ignored possible changes in the monsoon pattern for the period 2017–2028.

The aquifer parameters of the steady-state model were modified during manual calibration of the transient model, keeping the values within the ranges used by other workers for smaller-scale groundwater flow modelling in northwestern India^26,32,33^. Using a trial and error approach in several runs of the model, the calibration was mainly accomplished by varying the horizontal hydraulic conductivity (K_x_ and K_y_; see Table 2) in the range of 8 to 16 m/day, and specific yield in the range of 0.1 to 0.3. The final calibrated aquifer parameters given in Table 2 correspond to those of fine sand, which may be a suitable description of the approximate bulk characteristics of the regional aquifer^34,35^. Further, in order to improve the calibration statistics, the groundwater abstraction values were adjusted in the range of ± 20 percent.

**Table 2:** Calibrated aquifer parameters for the regional transient model.

Calibrated aquifer parameters	Values
K_x_ (Hydraulic conductivity along X-axis)	9 m/day
K_y_ (Hydraulic conductivity along Y-axis)	9 m/day
K_z_ (Hydraulic conductivity along Z-axis)	0.9 m/day
S_y_ (Specific yield)^35^	0.2

The calibrated transient model was used to generate a baseline scenario of future groundwater level change by running the model up to the year 2028, keeping the groundwater abstraction values constant at the level of 2017 for the period 2017–2028. The model was then run with a 20 percent reduction in groundwater abstraction in the study area for the period 2017–2028. As groundwater abstraction in the study area is primarily for agricultutual acitivty, this reduction was used as a proxy to assess the spatial and temporal impacts of the proposed improvement of 20 percent in agricultural groundwater use efficiency.

## Results and discussion

**Figure 9:**
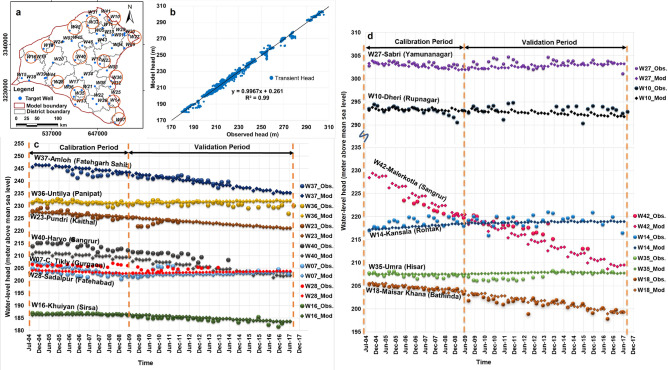
(**a**) Locations of the 46 representative CGWB observation/target wells; 13 of the wells for which observed and modelled water levels are shown in c and d are circled. (**b**) Plot of all 1786 available observed head values (January, May, August and November) versus model-generated head values (January, May, August and November) for the period July 2004 to August 2017. The linear regression line shows the results of calibration (2004–2009) and validation (2009–2017) of the groundwater flow model. c and d. Plot of observed (shown with suffix ‘Obs’) and model generated (shown with suffix ‘Mod’) water levels with respect to time for the calibration and validation periods.

The regional groundwater flow model was calibrated and validated for the period from July 2004 to August 2017. The transient model calibration was excellent (Fig. 9) and the water-table contours produced by the model at the end of calibration (Supplementary Fig. S7) and validation (Supplementary Fig. S8) were very similar to observations. Observed groundwater level variation in the 46 target wells closely matched the model estimates, with small residuals (Supplementary Fig. S9). The residual mean was estimated at 0.48 m, and the absolute residual mean was estimated at 1.48 m, which are quite reasonable. For better visual appreciation, we show in Fig. 9 the representative target wells with their hydrographs and the overall calibration statistics as a plot of the model generated head versus observed head.

**Figure 10:**
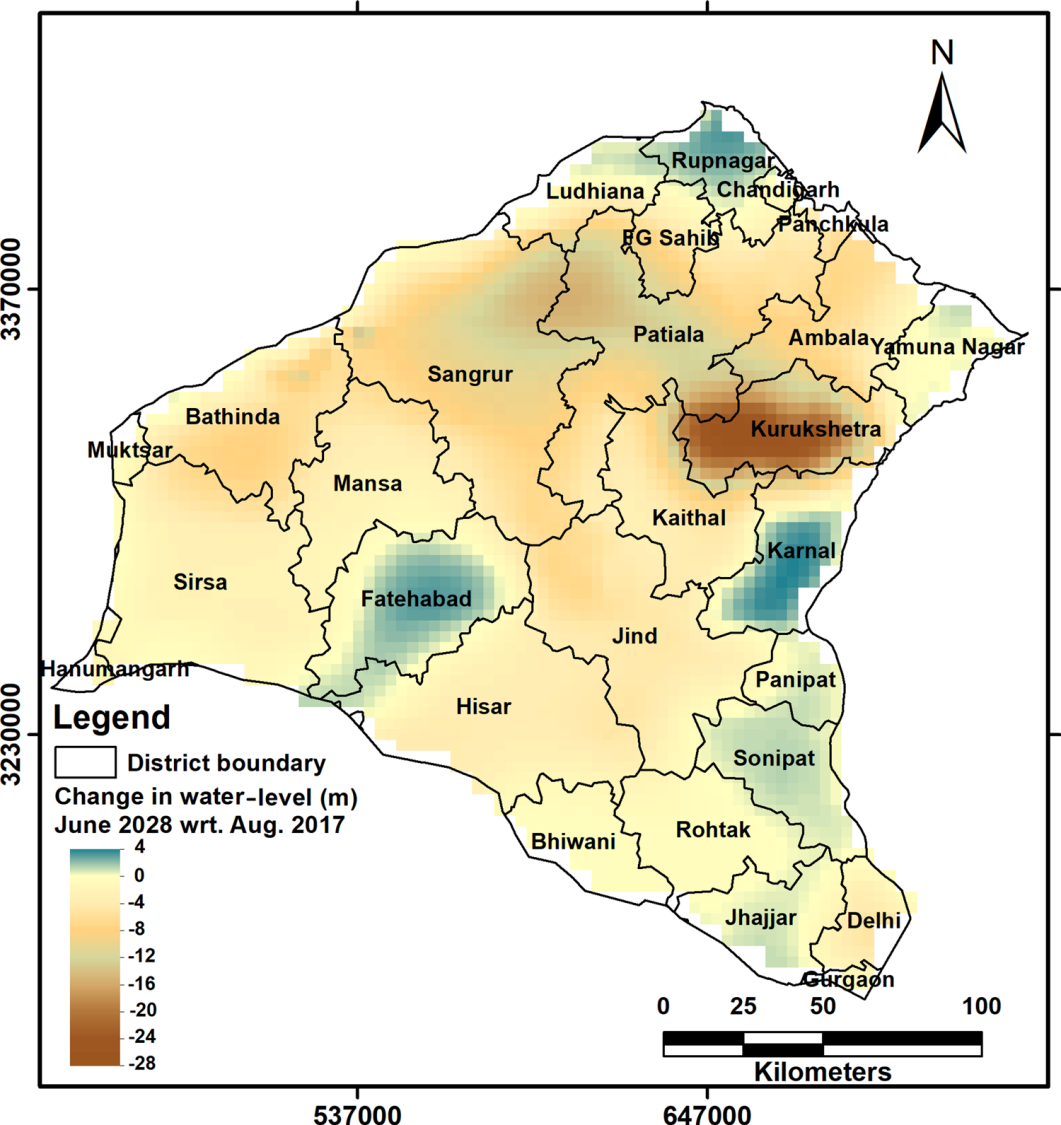
Map showing spatial variation in water-level change for June 2028 with respect to August 2017 in the baseline scenario with no mitigation measures.

The baseline model predicts future groundwater level trends up to June 2028, assuming that the level of groundwater abstraction remains the same for the period 2017–2028. For better visual appreciation, in each model grid cells, the last water-level head generated by the model for June 2028 was subtracted from the water-table head generated by the model for August 2017 at the end of the validation period (Fig. 10). This baseline scenario shows that a maximum decline of about 28 m would be observed in Kurukshetra district, with smaller declines in Patiala, Sangrur, Fatehgarh Sahib, and parts of Ambala district (Fig. 10). Other areas are predicted to show water-level declines in the range of 5–10 m. Areas in the southeastern part of the model space, including Sonipat, Panipat, Jhajjar, Bhiwani, and Rohtak districts, show either no change in water level or marginal rises of up to 1 m. As discussed earlier, this may be a manifestation of the use of canal water for irrigation in these localities. Other districts such as Rupnagar, Karnal and areas along the border of Fatehabad and Hisar district show greater rises in the water-level of up to 4 m. Except for Rupnagar, which is close to a barrage, these rising groundwater levels were not expected. We re-examined the observational data for these localities used for model calibration (Supplementary Figs. S10, S11, and S12).

**Figure 11:**
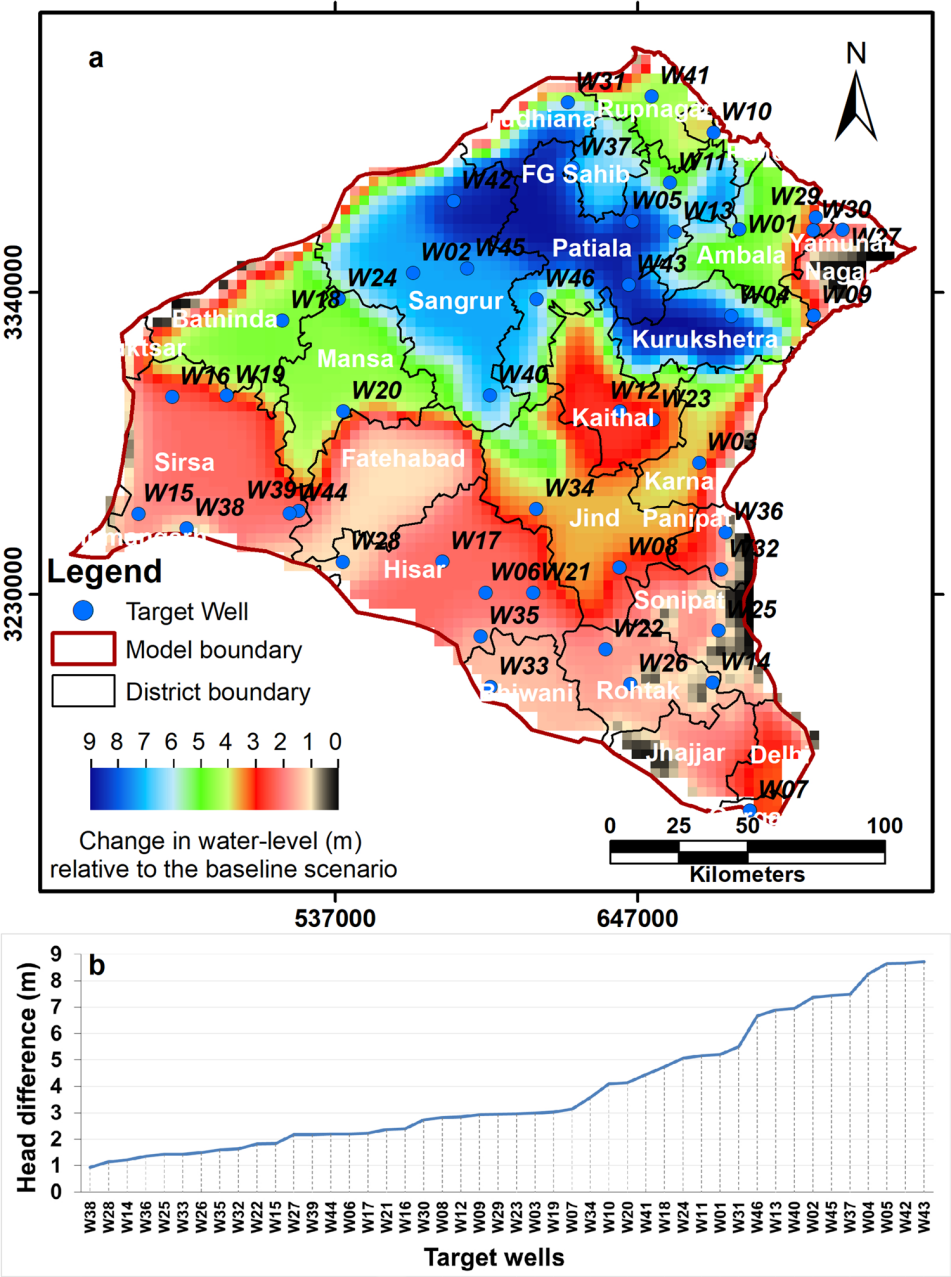
(**a**) Map showing spatial variation in the amount of rise in water-level in June 2028 in response to the scenario of 20 percent reduction in groundwater abstraction compared to the baseline scenario. (**b**) Plot of the water-level rise for the observation/target wells, ordered by the magnitude of the change between scenarios.

From 2010 onwards, the observed water levels in all three districts show a rising trend. The rising water-level trend in Karnal may be due to a decrease in groundwater abstraction for irrigation (compare groundwater abstraction values for irrigation from 2011 and 2013 in Table S1). Anecdotal evidence collected in the field suggests that this may be due to restrictions imposed by the Haryana state government on the sowing of paddy crops during the pre-monsoon season. The rising groundwater level at Fatehabad-Hisar border is more likely to be due to shallow salinity in groundwater, which restricts its use and forces the farmers to utilise canal water for irrigation. The model correctly simulates a declining water level for Karnal and Fatehabad-Hisar border area for 2004 to 2010, which reverses after 2010, while for Rupnagar it simulates a rising trend over the entire model run (Supplementary Figs. S10, S11 and S12). We then tested the efficacy of a single plausible demand-side management scenario of a 20 percent reduction in groundwater abstraction, in order to simulate a 20 percent increase in use efficiency. The mitigation scenario shows that the rate of groundwater decline decreases compared to the baseline scenario for the period 2017–2028. Specifically, the model results show that water levels are up to 9 m higher compared to the baseline scenario in 2028 (Fig. 11). The difference in the projected water level trends for the mitigation scenario with 20 percent reduction in groundwater abstraction for the period 2017–2028 compared to the baseline scenario with no change in groundwater abstraction for the same period can also be illustrated as hydrographs produced by the model (Supplementary Fig. S13). We observed the maximum positive impact in the critically overexploited districts of Kurukshetra, Patiala, Sangrur, Fatehgarh Sahib and parts of Ambala. Effectively, the 20 percent reduction in abstraction decreases the water-level decline rate from about 0.6–2.5 m/year at present to 0.2–1.6 m/year in these areas, a change of 36–67 percent. In contrast, there is little change in the rate of water-level decline in areas with shallow present-day groundwater levels and areas near the canal network. In these areas, limited groundwater abstraction and significant infiltration from canals, likely leads to less unsaturated space in the aquifer and hence there is less scope for changes in the water-level in response to reduced abstraction.

Studies of irrigation sustainability in the High Plains Aquifer in the central US have shown that even a modest reduction in pumping, comparable to the 20 percent reduction modelled here, can lead to effective moderation in groundwater decline^36–38^. Such a reduction can potentially be achieved by the adoption of more efficient irrigation methods as well as changes in crop intensity and pattern^17–20^. A number of studies have cautioned, however, that increased water use efficiency may not always result in water use reduction, because the water saved may be used for more water-intensive crops or to expand irrigated areas^2,39^. Cao et al. (2013), in a similar regional groundwater model-based assessment for mitigation of the groundwater crisis of the North China plain, emphasised the importance of water use efficiency coupled with other strategies such as artificial recharge in designated areas^1^. In this context, a detailed strategy for improvement in irrigation water use efficiency that results in actual water use reduction must be designed and implemented, in close collaboration with stakeholders.

## Limitations and scope for future study

The model presented here is a simplification of an extensive, complex regional aquifer system. This simplification is justified in part because the system comprises spatially-heterogeneous but regionally-developed alluvial sediments deposited by the Indus and Ganga river networks ^8,11^, with broad hydrogeological similarity, thereby requiring a similar groundwater development and management strategy^40,41^. The value of this approach is to provide a synoptic, system-scale view of the response to large-scale changes in abstraction. The assumption of a homogeneous, anisotropic aquifer is not appropriate; however, for investigation of more local problems, the inclusion of local heterogeneity in model parameters is desired. The model also suffers from missing or incomplete input data and parameter values. The limitations of our approach provide scope for several potential improvements. We have used district-averaged recharge values which combine recharge from precipitation with recharge from other sources such as canal seepage and irrigation return flow. These recharge estimates were not available for all years, and so recharge values for 2004 were used for the entire simulation. Ideally, recharge from precipitation should have been estimated for every year from yearly precipitation data at a sub-district scale and incorporated in the model along with a yearly estimate of recharge from other sources. It would be desirable in future to calibrate the model with recharge estimates based on precipitation, which may vary in both space and time.

In the present study, we have calibrated the model in part by allocating the available groundwater abstraction estimates across different 2-month stress periods, using the observed cropping pattern in the region, and by adding a small additional abstraction component in the towns to cover domestic use. Such indirect inference of groundwater abstraction from cropping pattern has inbuilt uncertainty, which introduces uncertainty in the model output. Future modelling efforts should be informed by extensive pumping data which may require registration of each tubewell and the use of flow meters, similar to the approach that has been used in the High Plains Aquifer^37^. An improved future model should incorporate spatial heterogeneity in specific yield and hydraulic conductivity, perhaps estimated using long-duration pumping tests, rather than relying on literature-based average values. To better assess the proposed reduction in groundwater abstraction, specific yield estimates could be complemented and verified using a water balance approach for better representation of the aquifer system being modelled^36–38^. In future, if the Ghaggar River discharge data is available, then the river could be correctly simulated as a river boundary in the model.

Finally, this study did not consider evapotranspiration separately; instead, groundwater loss due to evapotranspiration was factored into the total groundwater abstraction. Explicit incorporation of both precipitation and evapotranspiration would allow investigation of the potential impacts of future changes in precipitation amounts and pattern, as well as changes in cropping patterns or land use, on the regional groundwater flow model.

## Conclusions

The groundwater system of northwestern India provides food security to India, and the current groundwater over-exploitation crisis in this region is a pressing concern. We have presented regional-scale transient simulations of the groundwater system for the period 2004–2028 under two different scenarios: 1. A baseline scenario in which variation in groundwater abstraction was incorporated in the model for the duration 2004 to 2017, while abstraction for 2017 to 2028 remains constant at 2017 levels, and 2. A mitigation scenario, which retains the variation in groundwater abstraction for the duration 2004 to 2017, where as a mitigation measure, the groundwater abstraction for the period 2017–2028 is reduced by 20 percent. Our modelling reveals that, if the present level of groundwater abstraction continues at the same level until 2028, groundwater levels will decline at rates of up to 2.8 m/year in critically overexploited areas like Kurukshetra, Patiala and Sangrur districts. In contrast, a reduction in groundwater abstraction by 20 percent has the potential to generate a significant positive impact on groundwater levels within a decade. It could retard the rate of groundwater decline by up to 67 percent in areas that are currently overexploited. However, the magnitude of the impact varies spatially based on the current levels of abstraction and recharge. Comparison of our results to studies of other critically overexploited aquifer systems suggests that an integrated approach to agricultural water management practice, incorporating groundwater use efficiency, water-efficient cropping intensity, pattern, and rainwater harvesting for agricultural use, is strongly advised.

## Supplementary information

Supplementary Information.
