# Supplementary material for: Modelling water levels of northwestern India in response to improved irrigation use efficiency
Source: Sci Rep. 2020 Aug 10;10:13452. doi: 10.1038/s41598-020-70416-0 (PMC7417598; doi:10.1038/s41598-020-70416-0)
Supplement: Supplementary file 1 — Supplementary Information. [file 41598_2020_70416_MOESM1_ESM.pdf]

Supplementary Information

Modelling water levels of northwestern India in response to improved irrigation use efficiency

[1+*]Shashank Shekhar, [1&2+]Suman Kumar, [3]A. L. Densmore, [3&4]W. M. van Dijk, [2]Rajiv Sinha, [1&5]Manoranjan Kumar, [2]Suneel Kumar Joshi, [6]Shive Prakash Rai, [7]Dewashish Kumar

[+] Equal contributors; [*]for correspondence: shashankshekhar01@gmail.com

[1]Department of Geology, University of Delhi, Delhi-110007, India.

[2]Department of Earth Sciences, Indian Institute of Technology, Kanpur-208016, India.

[3]Institute of Hazard, Risk, and Resilience and Department of Geography, Durham University, Durham DH1 3LE, UK.

[4]Water & Environment Division, Arcadis, Piet Mondriaanlaan 26, 3812 GV Amersfoort, The Netherlands.

[5]Geological Survey of India, Jaipur, India.

[6]Department of Geology, Banaras Hindu University, Varanasi, India.

[7]NGRI, Uppal Road, Hyderabad -500007, India.

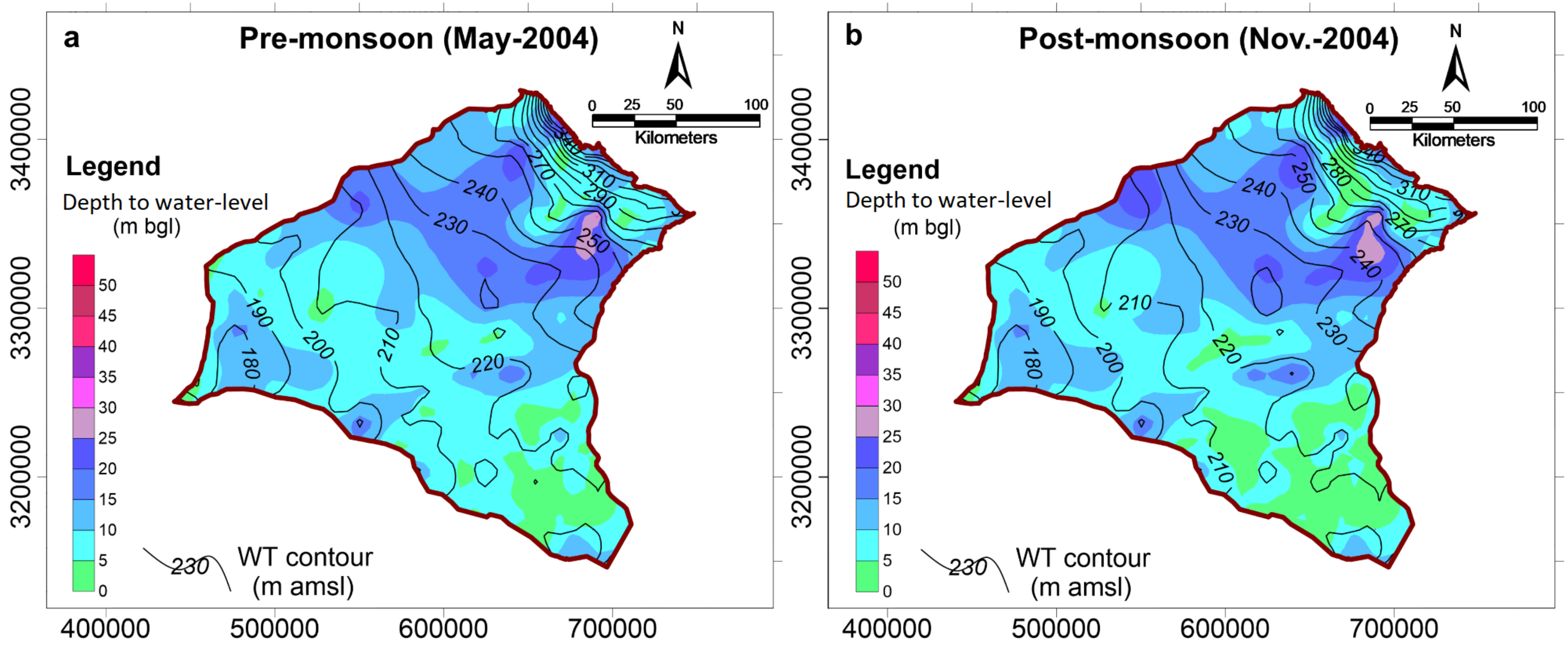

Fig.S1. Map showing pre and post-monsoon water table (WT) contours of 2004 for the study area. The colour variations shown as legend represent variation in the depth to water-level. (m amsl: meters above mean sea level).

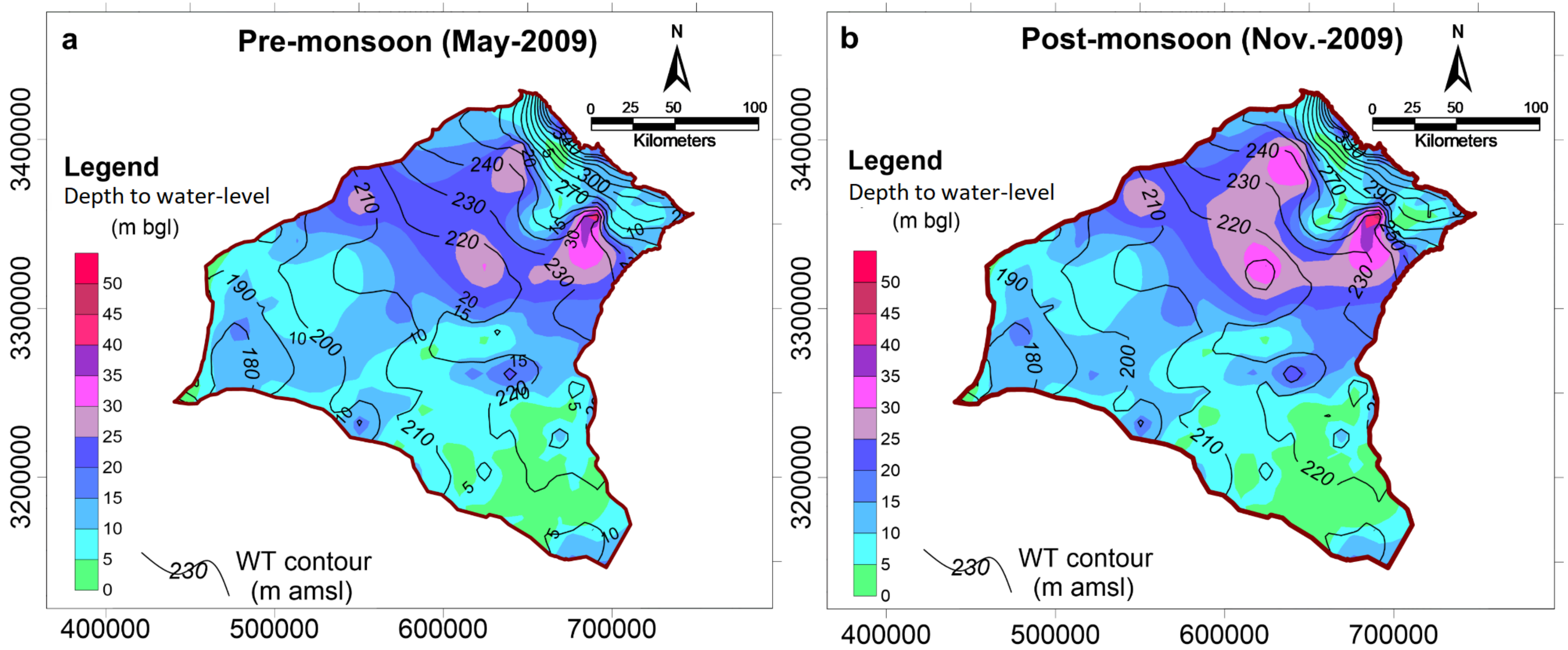

Fig.S2. Map showing pre and post-monsoon water table (WT) contours of 2009 for the study area. The colour variations shown as legend represent variation in the depth to water-level. (m amsl: meters above mean sea level).

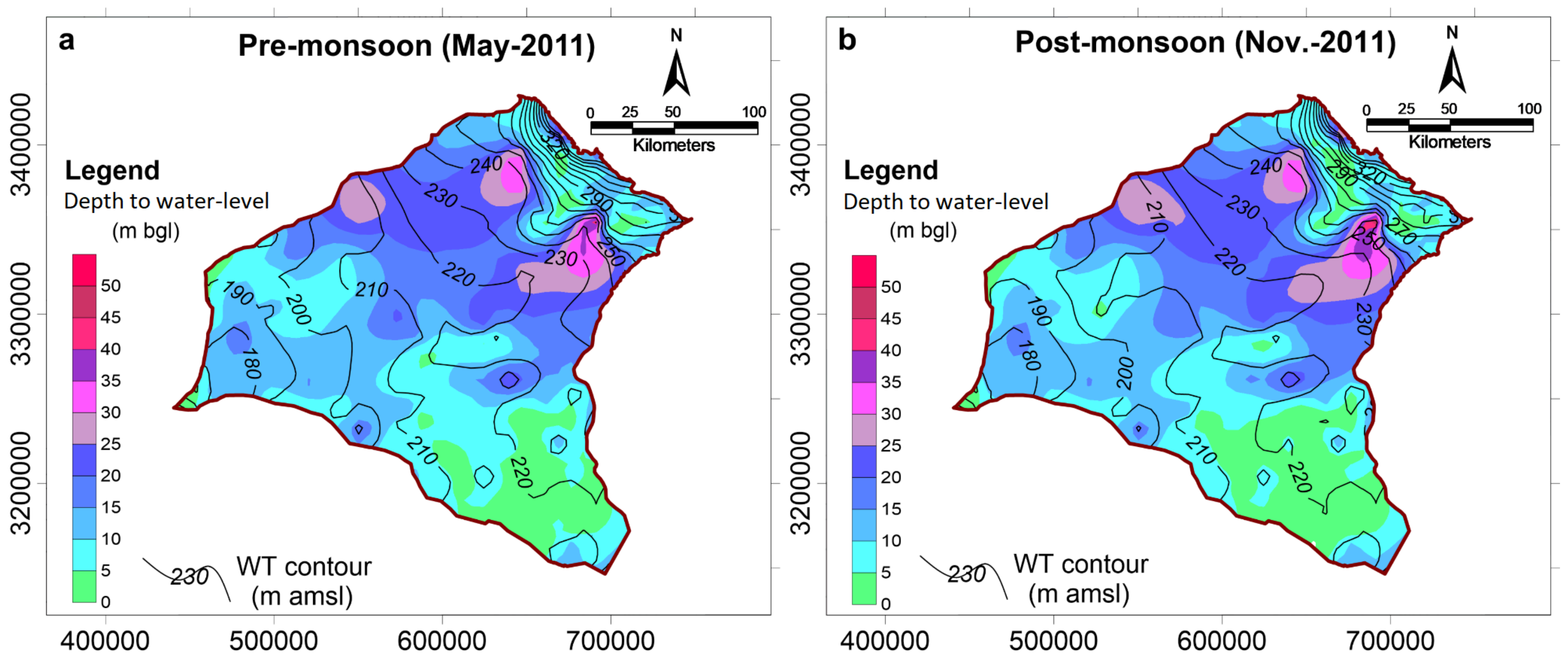

Fig.S3. Map showing pre and post-monsoon water table (WT) contours of 2011 for the study area. The colour variations shown as legend represent variation in the depth to water-level. (m amsl: meters above mean sea level).

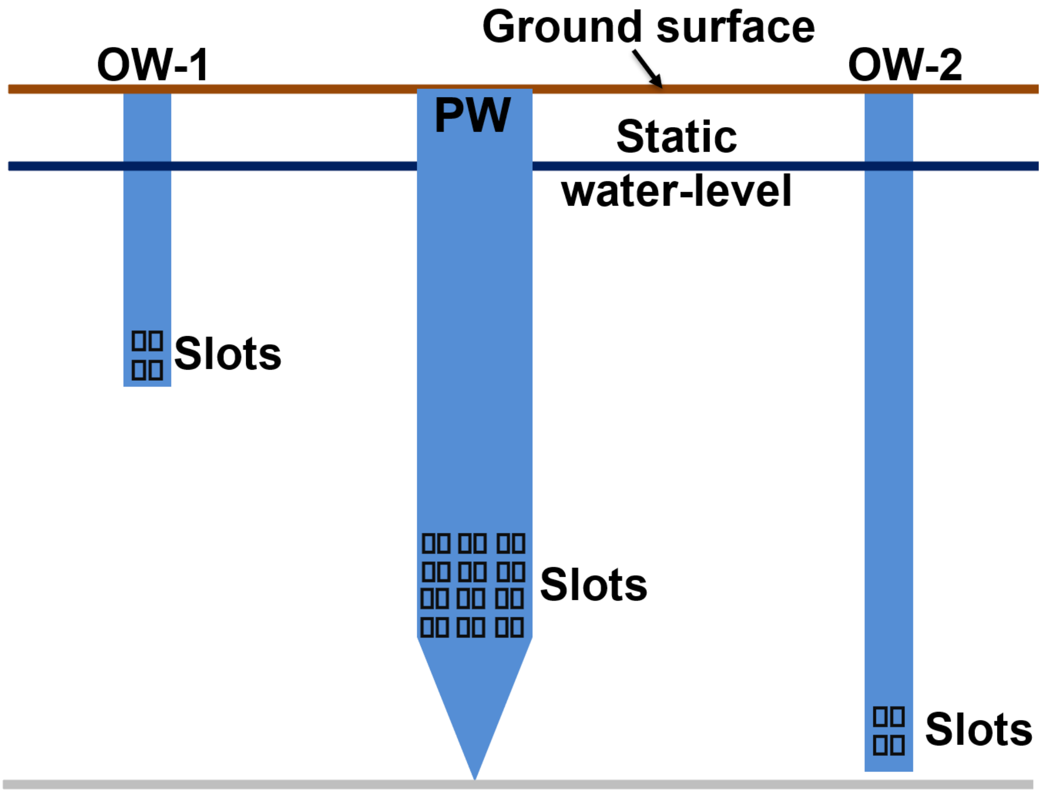

Fig.S4. Site plan of the experimental investigation site. Here OW-1 is the observation well-1 ; OW-2 is the observation well-2 and PW is the pumping well.

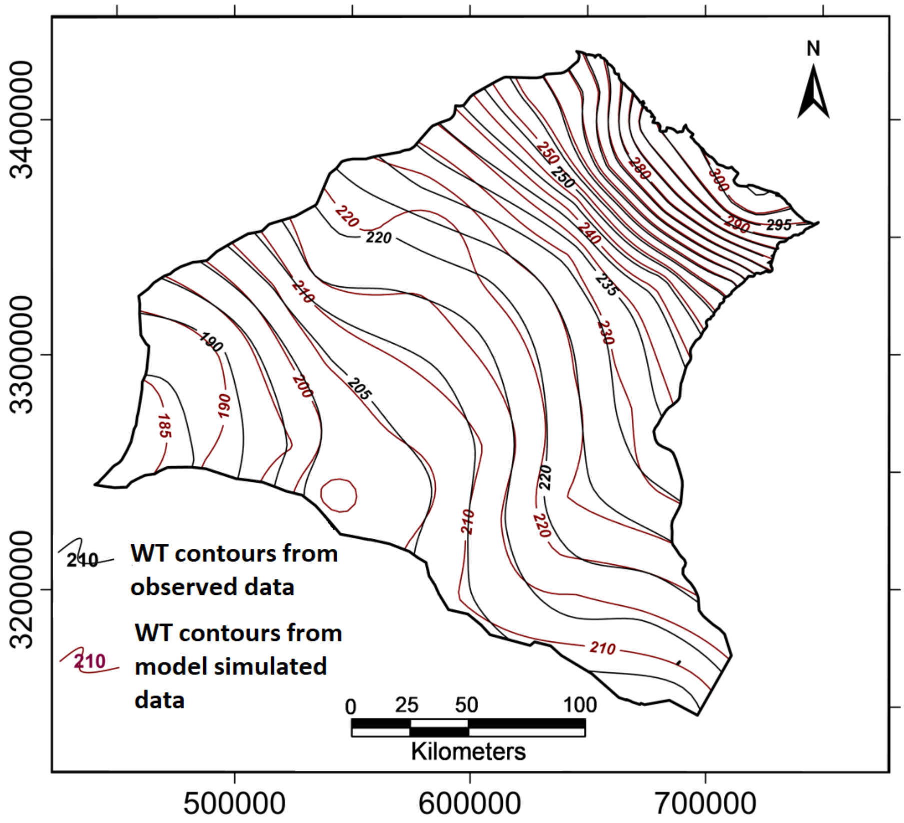

Fig.S5. Map showing the water table (WT) contours prepared from the observed data and the steady-state model simulated/generated data for August 2004.

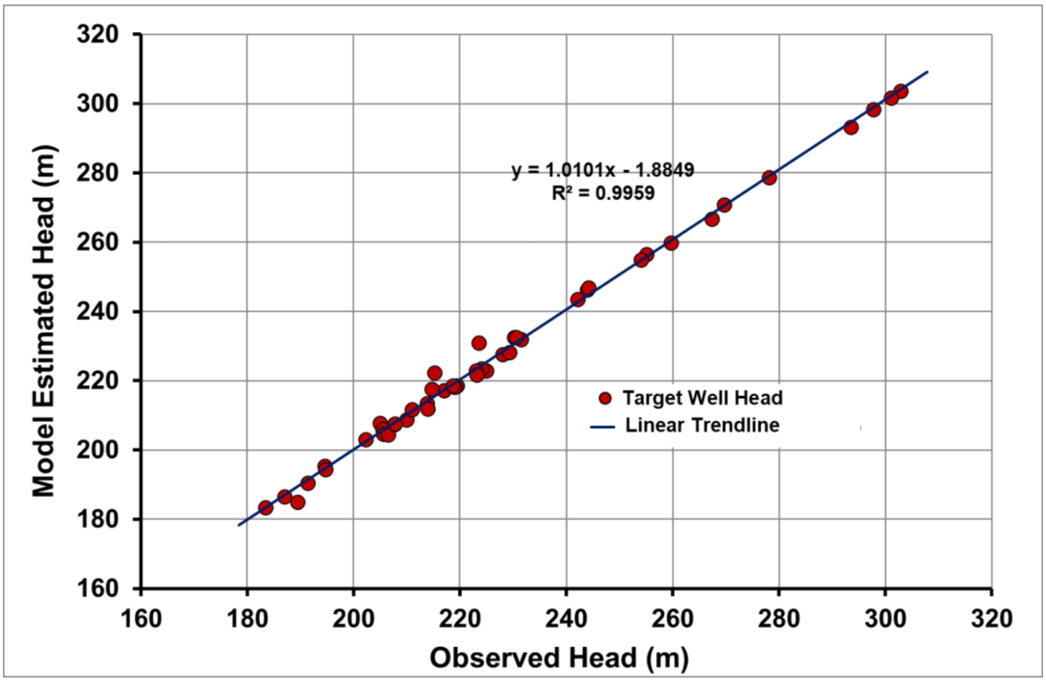

Fig.S6. Plot of model estimated head versus observed head showing calibration of the steady state model.

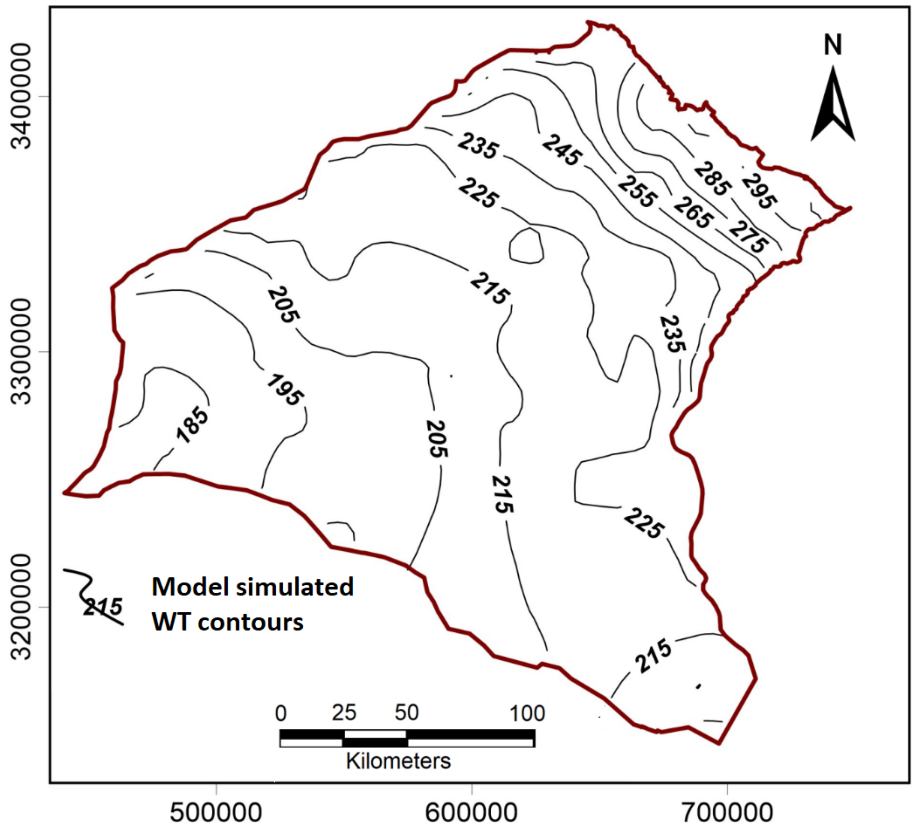

Fig.S7. Model simulated water table (WT) contour map for June 2009 (30th stress period).

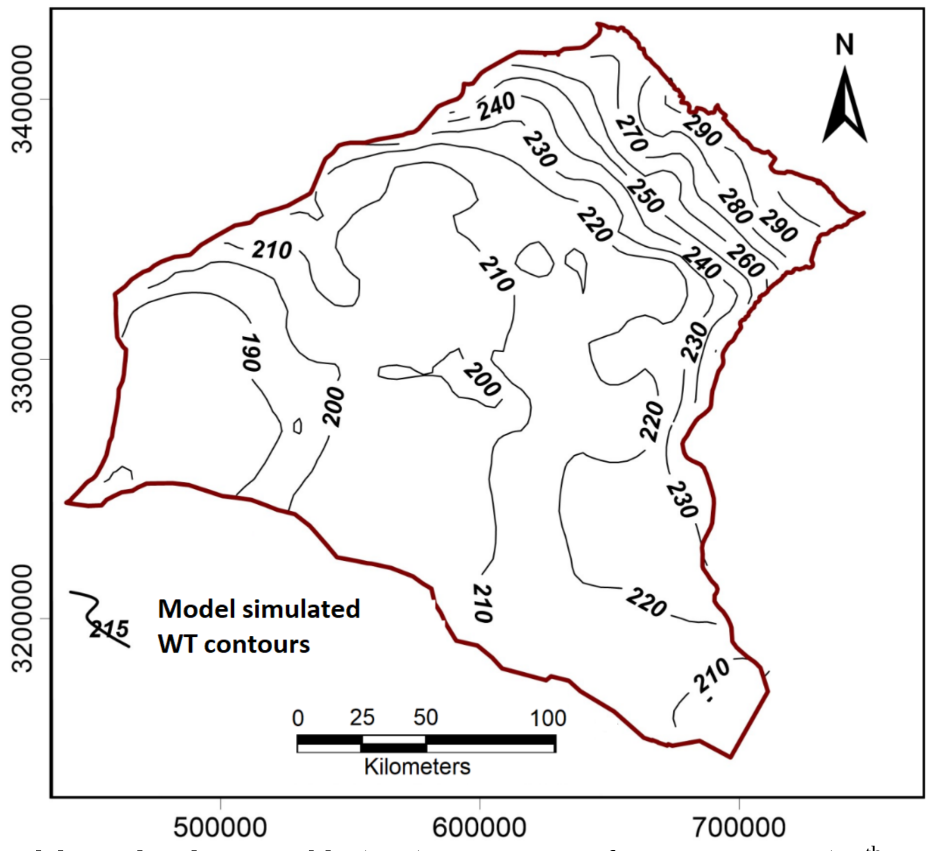

Fig.S8. Model simulated water table (WT) contour map for August 2017 (79th stress period).

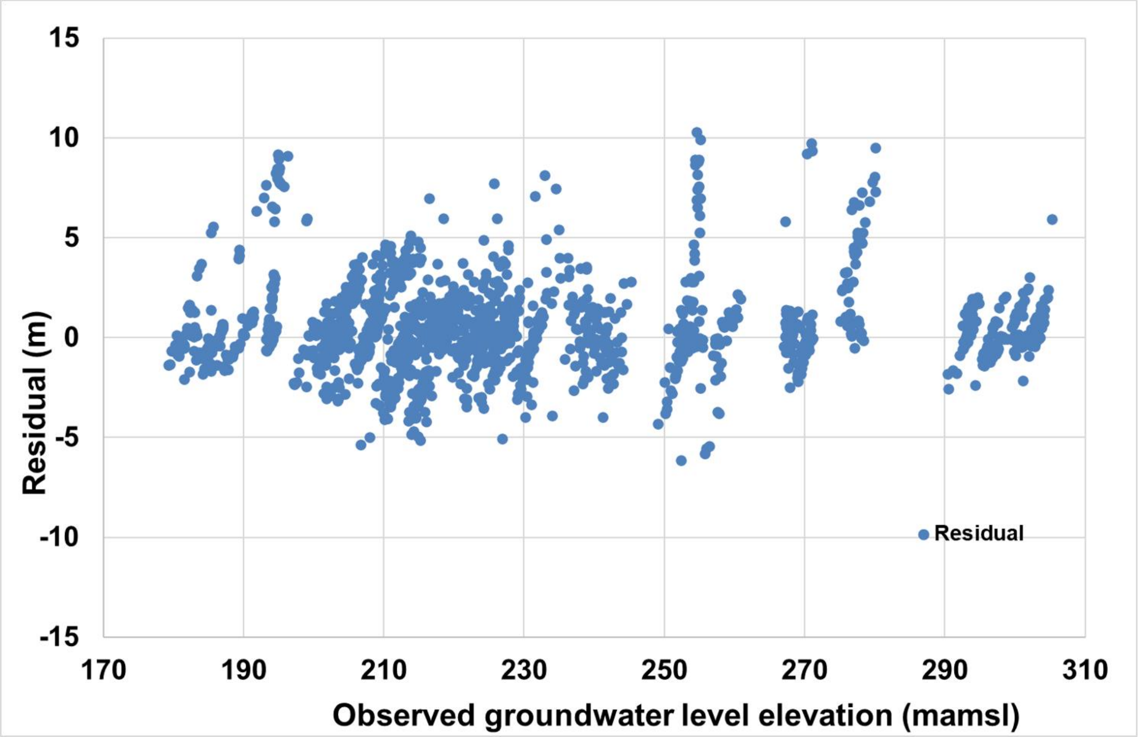

Fig.S9. Plot of observed minus simulated water-level elevation (Residual) versus observed water level elevation. (mamsl: meters above mean sea level)

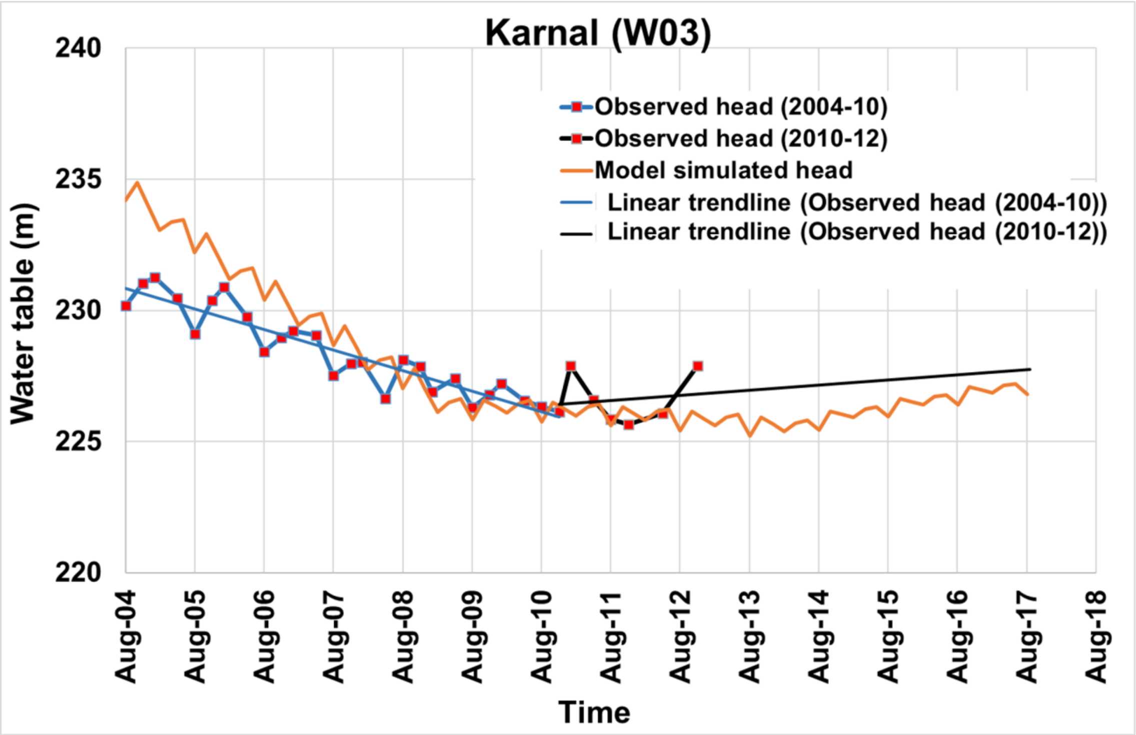

Fig.S10. Groundwater level hydrograph for the observation well at Karnal showing declining trend of water-level for 2004-10, stabilization and rising trend of the water-level after 2010.

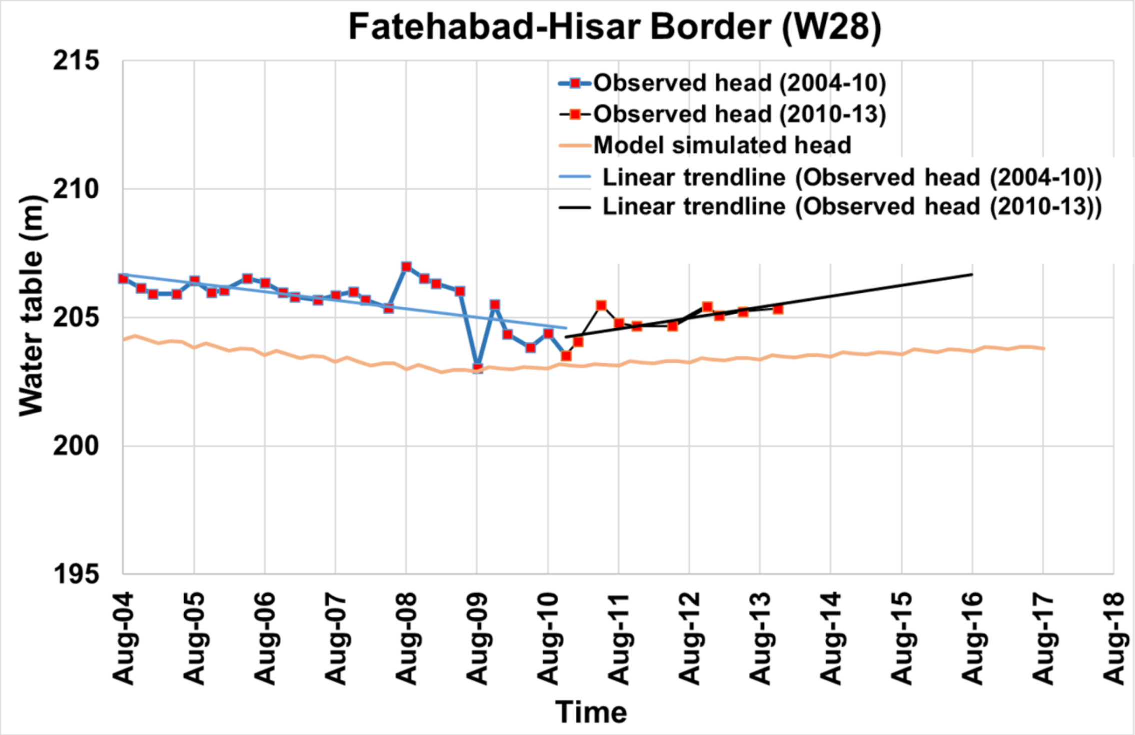

Fig.S11. Groundwater level hydrograph for the observation well at Fatehabad-Hisar border showing declining trend of water-level for 2004-10, stabilization and rising trend of the water-level after 2010.

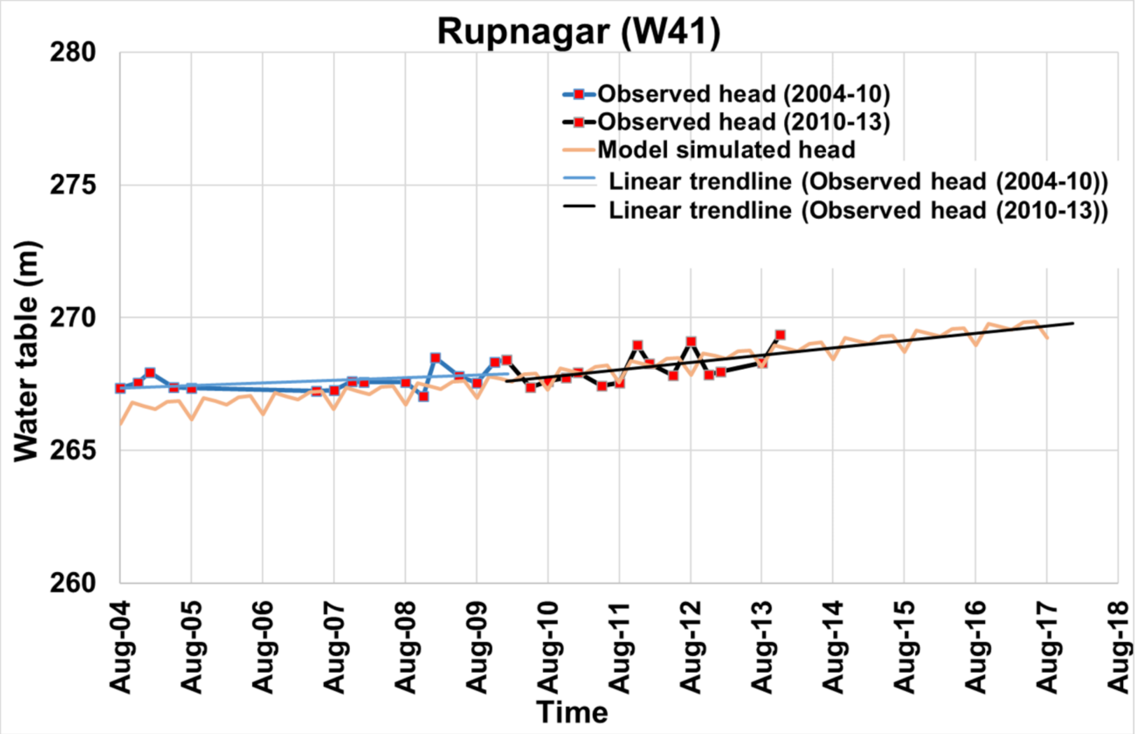

Fig.S12: Groundwater level hydrograph for the observation well at Rupnagar showing rising trend in the water levels.

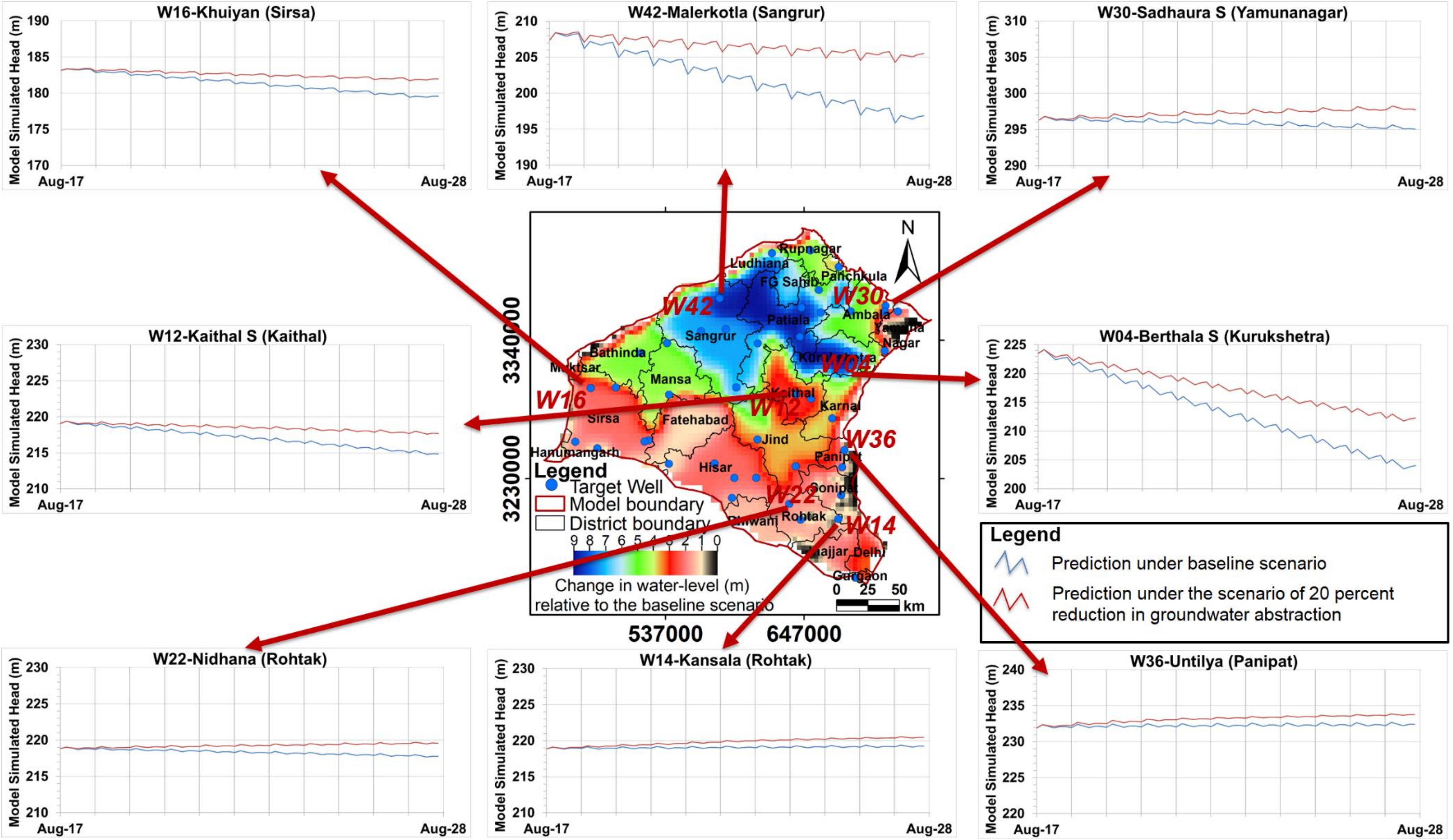

Fig.S13. A synoptic view of the predicted water-level trends for the representative groundwater level monitoring stations; for the baseline scenario and for the scenario with 20 percent reduction in the groundwater abstraction.

Table S1: Groundwater abstraction for Irrigation in years 2004, 2009, 2011 and 2013 corresponding to the districts comprising the study area[1-4].

Zone No.	State	District	Cultivable Area (*10 ^{6}m^2)$	Annual groundwater abstraction for irrigation (*10 ^{4}m^3)				Allocation of groundwater abstraction (%)	
				Year-2004	Year-2009	Year-2011	Year-2013	Monsoon	Non-Monsoon
1	Punjab	ROPAR	780	47717	43487	43775	43686	65	35
2	Punjab	LUDHIANA	3060	323274	334616	336828	333230	73	27
3	Punjab	FATEHGARH SAHIB	1020	83552	110450	111180	109995	71	29
4	Punjab	PATIALA	2706	264951	286960	288631	285530	76	24
5	Punjab	SANGRUR^	4355	414055	482667	485274	480635	75	25
6	Punjab	BATHINDA	2970	78412	117778	118446	130255	64	36
7	Punjab	MANSA	1900	140412	143790	144741	143197	55	45
8	Haryana	SIRSA	3900	66646	115634	135443	110751	45	55
9	Haryana	AMBALA	1320	33975	41583	38323	49756	45	55
10	Haryana	YAMUNANAGAR	1250	48333	55077	56434	58647	45	55
11	Haryana	KURUKSHETRA	1500	64196	67904	71099	135122	45	55
12	Haryana	KAITHAL	2020	98154	101504	108316	114114	45	55
13	Haryana	KARNAL	2201	119235	118899	120647	85311	45	55
14	Haryana	JIND	2350	46820	77363	91079	111148	45	55
15	Haryana	FATEHABAD	2250	52898	107316	90660	115758	45	55
16	Haryana	HISSAR	3400	37456	59836	57841	77990	45	55
17	Haryana	SONEPAT	1580	48179	90622	102617	86500	45	55
18	Haryana	ROHTAK	1420	16790	28446	30488	33285	45	55
19	Haryana	BHIWANI	3950	59307	43068	70596	103397	45	55
20	Haryana	JHAJJAR	1590	30873	40751	40768	35002	45	55
21	Delhi	Delhi	452.71 [5]	20002	14024	14024	13763.5	25	75
22	Haryana	GURGAON	760	51157	35777	36266	28062	45	55
23	Haryana	PANIPAT	940	50939	50961	50148	53347	45	55
24	Rajasthan	HANUMANGARH	7944	15699	15627	15716	13355.9	25	75
25	Punjab	MUKTSAR	2240	51990	51286	51625	51061	73	27
26	Haryana	PANCHKULA	240	7731	9072	8464	8862	45	55

$ Department of Agriculture, Cooperation &Farmer Welfare (Ministry of Agriculture & Farmers Welfare, Government of India) http://agricoop.nic.in/agriculture-contingency-plan-listing . ^ Combined area under cultivation of Sangrur and Barnala districts.

Table S2: The design parameters of the observation and pumping well at the site of the experimental investigation.

**Shallow observation well (OW-1)**		**Deep observation well (OW-2)**		**Pumping well (PW)**	
Drilling Depth	Slotted pipes (Diameter: 0.1 m)	Drilling Depth	Slotted pipes (Diameter: 0.1 m)	Drilling Depth	Slotted pipes (Diameter: 0.25 m)
50 m	40-44 m	91 m	87-91 m	158 m	76-86 m
At 10 meter south west from PW		At 45 meter north east from PW		Pumped at discharge of 288 m^3/day	

Table S3: Monsoon and non-monsoon recharge for 2004, 2009, 2011 and 2013 corresponding to the districts comprising the study area[1-4].

Recharge Zone No	State	District	Geo. Area (*10 ^{6}m^{2})$	Monsoon Recharge(*10 ^{4}m^{3})				Non-Monsoon Recharge (*10 ^{4}m^{3})			
				Year-2004	Year-2009	Year-2011	Year-2013	Year-2004	Year-2009	Year-2011	Year-2013
1	Punjab	ROPAR	1440	44318	31716	32081	32316	20844	13574	13542	13602
2	Punjab	LUDHIANA	3860	181540	153524	158825	176954	78590	72529	72441	59350
3	Punjab	FATEHGARH SAHIB	1147	42717	46163	46683	51780	15690	13287	13227	13483
4	Punjab	PATIALA	3290	138654	123503	123995	131665	42555	42144	42062	38455
5	Punjab	SANGRUR^	5020	187158	171507	163322	197735	66416	49518	47223	63415
6	Punjab	BATHINDA	3367	58865	61834	64964	91092	34182	45606	46174	66907
7	Punjab	MANSA	2171	59017	50619	53271	73300	30339	24099	24115	41610
8	Haryana	SIRSA	4277	29097	38821	42468	34448	32744	40602	43478	36304
9	Haryana	AMBALA	1574	39004	40276	41757	43457	15524	16570	16344	17958
10	Haryana	YAMUNANAGAR	1756	39490	36485	36696	37742	17316	17070	17125	18246
11	Haryana	KURUKSHETRA	1530	28929	25617	27045	41033	14305	11143	11326	16413
12	Haryana	KAITHAL	2317	41737	36991	38237	38905	20832	19434	20465	20546
13	Haryana	KARNAL	2520	61785	53334	53131	43394	31687	37998	37112	34675
14	Haryana	JIND	2702	39066	42813	47673	55798	36342	43201	45868	56730
15	Haryana	FATEHABAD	2490	25038	41398	37993	41528	16182	22395	20886	28726
16	Haryana	HISSAR	4050	33010	38780	38327	42855	27863	30954	30591	33985
17	Haryana	SONEPAT	2122	26715	45854	47677	50707	20608	35646	37876	37813
18	Haryana	ROHTAK	1745	15441	25872	26132	29844	12106	21514	21832	23261
19	Haryana	BHIWANI	5140	43710	40385	46486	43299	18304	18622	21598	25524
20	Haryana	JHAJJAR	1834	19867	25226	25586	27828	18516	19742	19721	19351
21	Delhi	Delhi	1466 [5]	18714	20969	20969	10964.5	10996	10083	10082	22940.3
22	Haryana	GURGAON	1254	32300	14751	15583	15364	15345	11095	11137	11111
23	Haryana	PANIPAT	1268	23264	21173	21501	22727	12869	12665	13038	14252
24	Rajasthan	HANUMANGARH	9656.1	9488	9888	9904	7940.61	11842	12670	12745	10318.8
25	Punjab	MUKTSAR	2630	58963	47956	49190	49104	33695	37388	37381	35478
26	Haryana	PANCHKULA	898	10717	10193	10537	10858	4940	4413	4361	4710

$ CGWB, http://cgwb.gov.in/District-GW-Brochures.html
^ Combined area of Sangrur and Barnala districts.

Table S4: The observed groundwater table elevation for January, May, August, and November months during the calibration and validation period of July 2004 - August 2017.

Target Well	State/UT	District	Block/Taluka	Village/Location	Lat.	Long.	Surface Elevation
W01	Punjab	Patiala	Dera Bassi	Antala	30.38	76.91	280.8
W02	Punjab	Sangrur	Barnala	Badbar	30.25	75.67	232.4
W03	Haryana	Karnal	Assandh	Bala	29.6	76.78	241.2
W04	Haryana	Kurukshetra	Babain	Berthala S	30.12	76.98	271.3
W05	Punjab	Patiala	Patiala	Birkauli	30.41	76.51	261.4
W06	Haryana	Hisar	Hansi	Chanaut	29.2	75.92	214.1
W07	Haryana	Gurgaon	Farukhnagar	Chandu Tikly	28.47	76.91	213.7
W08	Haryana	Jind	Jind	Chhabri	29.26	76.45	228.6
W09	Haryana	Yamunanagar	Jagadhri	Dhaurang M	30.06	77.19	275.3
W10	Punjab	Rupnagar	Kharar	Dheri	30.62	76.69	298.4
W11	Punjab	Patiala	Rajpura	Haluka	30.55	76.65	277.7
W12	Haryana	Kaithal	Kaithal	Kaithal S	29.8	76.4	237.6
W13	Punjab	Patiala	Ghanaur	Kami Kalan	30.37	76.67	267.3
W14	Haryana	Rohtak	Rohtak	Kansala	28.89	76.77	222.5
W15	Haryana	Sirsa	Elllenabad	Kash Ram Dhab	29.42	74.61	192.6
W16	Haryana	Sirsa	Dabwali	Khuiyan	29.85	74.76	197.9
W17	Haryana	Hisar	Barwala	Kirori	29.3	75.8	216.6
W18	Punjab	Bathinda	Maur	Maisar Khana	30.1	75.18	214.2
W19	Haryana	Sirsa	Odhan	Mithri	29.85	74.98	202.8
W20	Punjab	Mansa	Budhlada	Mofar	29.77	75.41	214.9
W21	Haryana	Hisar	Narnaud	Mothmajri	29.2	76.09	220.1
W22	Haryana	Rohtak	Meham	Nidhana	29.01	76.38	222
W23	Haryana	Kaithal	Pundri	Pundri	29.75	76.56	242.2
W24	Punjab	Mansa	Bhikhi	Ralla	30.12	75.44	224.9
W25	Haryana	Sonipat	Gohana	Rithal Phogat	28.99	76.76	225.1
W26	Haryana	Rohtak	Rohtak	Rohtak	28.88	76.58	217.9
W27	Haryana	Yamunanagar	Sadhura	Sabri	30.35	77.24	309.4
W28	Haryana	Hisar-Fatehabad border	Bhattu Kalan	Sadalpur	29.3	75.41	212.3
W29	Haryana	Yamunanagar	Sadhura	Sadhaura	30.4	77.22	307.4
W30	Haryana	Yamunanagar	Sadhura	Sadhaura S	30.38	77.21	302.6
W31	Punjab	Ludhiana	Machhiwara	Samrala 2(s)	30.88	76.2	265
W32	Haryana	Panipat	Israna	Shahpur	29.25	76.8	229.5
W33	Haryana	Bhiwani	Tosham	Tosham	28.87	75.91	217.7
W34	Haryana	Jind	Uchana	Uchana	29.46	76.18	229.7
W35	Haryana	Hisar	Hansi	UMRA	29.05	75.91	214.5
W36	Haryana	Panipat	Madlauda	Untilya	29.39	76.85	234.3
W37	Punjab	Fatehgarh Sahib	Amloh	Amloh	30.6	76.26	263.5
W38	Haryana	Sirsa	Elllenabad	Chilkani Dhab	29.39	74.8	196.3
W39	Haryana	Sirsa	Nathusari Chopra	Ding	29.46	75.26	207.3
W40	Punjab	Sangrur	Andana	Haryao	29.83	75.87	223.5
W41	Punjab	Rupnagar	Morinda	Kakrali	30.83	76.52	287.2
W42	Punjab	Sangrur	Malerkotla- I	Malerkotla	30.52	75.9	247.4
W43	Punjab	Patiala	Bhuner Heri	Mirapur	30.2	76.49	250.9
W44	Haryana	Sirsa	Nathusari Chopra	Sherpura	29.45	75.2	209.1
W45	Punjab	Sangrur	Bhawanigarh	Akbar Pur M	30.34	75.98	244.5
W46	Punjab	Patiala	Samana	Bhamna	30.15	76.13	240.9

Table S4

Target Well	Aug_ 2004	Nov_ 2004	Jan_ 2005	May_ 2005	Aug_ 2005	Nov_ 2005	Jan_ 2006	May_ 2006	Aug_ 2006	Nov_ 2006	Jan_ 2007
**W01**	278.08	278.44	278.16	277.16	277.81	277.89	277.74	276.7	276.56	276.66	276.56
**W02**	219.36	219.08	218.63	219.61	218.64	219.06	218.66	219	217.62	217.91	218.11
**W03**	230.18	231.04	231.25	230.46	229.1	230.38	230.88	229.75	228.44	228.97	229.21
**W04**	242.09	244.64	245.33	243.77	241.59	242.91	244.2	241.69	240.1	240.04	240.81
**W05**	243.95	243.6	243.89	243.4	242.16	242.52	242.4	242.1	239.15	241.54	240.8
**W06**	207.61	208.02	207.92	208.13	209.05	209.29	208.65	209.15	209.64	209.54	208.73
**W07**	205	204.51	203.5	203.53	203.55	203.65	202.95	202.5	203.01	201.9	201.85
**W08**	224.99	224.32	223.91	223.6	223.76	227.78	226.61	224	225.77	224.05	224.3
**W09**	259.7	260.5	260.89	259.77	259.44	260.26	260.48	259.5	258.49	259.16	259.18
**W10**	293.55	294.5	294.18	292.97	293.64	294.1	293.8	293.1	293.44	293.76	293.51
**W11**	269.68	271.01	270.86	269.13	269.08	269.08	270.53	271.05	270.83	270.71	270.62
**W12**	223.98	223.88	224.26	223.99	223.49	223.76	223.95	223.7	223.05	222.87	223.08
**W13**	255.105	255.5	255.45	255.3	254.97	254.53	254.5	254.03	253.69	253.3	253.1
**W14**	217.03	217.84	217.65	217.82	218.97	219.27	218.94	218.09	219.89	219.64	218.97
**W15**	183.37	183.39	183.1	183.32	183.04	183	182.84	182.89	183.1	182.85	182.7
**W16**	187.01	186.97	186.86	186.85	186.73	186.88	186.98	187	187.1	187	187.04
**W17**	202.3	202.46	202.37	202.17	202.55	202.92	202.52	202.5	201.65	203.15	202.75
**W18**	205.46	205.28	205.11	205	204.81	204.82	204.76	204.86	204.6	204.63	204.66
**W19**	191.35	191.43	191.23	191.2	191.17	191.3	191.26	191.14	191.25	191.5	191.25
**W20**	205.6	205.03	206.18	206.9	204.98	205.85	206.4	205.81	201.8	205.35	205.55
**W21**	213.73	215.27	214.89	214.09	214.17	216.28	215.57	214.45	213.19	214.73	214.23
**W22**	218.98	220.08	219.85	218.7	219.75	219.85	219.75	220.16	220.2	220.1	220.12
**W23**	228	228.56	229.21	229.32	227.44	227.62	228.17	228.32	226.42	225.87	226.39
**W24**	218.605	218.3	218.26	218.25	217.83	218.13	218.2	218.02	217.88	217.74	217.55
**W25**	222.99	223.17	223.08	222.75	223.07	223.38	223.6	223.35	224.05	222.35	222.57
**W26**	214.715	215.16	214.9	214.31	215.98	216.03	215.6	214.81	215.34	215.4	215.16
**W27**	302.87	303.66	303.44	302.99	303.9	303.83	303.57	303.04	303.73	303.43	303.06
**W28**	206.53	206.15	205.92	205.91	206.43	205.98	206.08	206.52	206.37	205.97	205.81
**W29**	301.12	301.7	301.66	301.35	301.05	301.01	300.78	300.56	300.34	300.12	299.9
**W30**	297.67	298.55	298.28	297.31	297.95	297.59	297.61	296.53	297.1	296.84	296.79
**W31**	254.06	254.26	253.47	253.42	253.57	253.58	253.48	252.68	252.18	252.36	252.24
**W32**	229.3	228.55	228.16	226.26	226.16	228.57	228.52	227.6	228.84	228.44	227.91
**W33**	209.88	210.28	209.3	209.55	210.05	210.22	209.75	211.44	209.58	209.08	207.95
**W34**	210.94	208.86	206.78	211.34	213.9	212.58	211.71	210.84	207.98	211.05	214.12
**W35**	207.78	207.94	207.36	207.13	208.07	208.15	207.57	206.99	207.43	207.13	206.53
**W36**	231.43	232.57	232.03	230.8	232.1	232.8	232.43	231.03	232.53	231.82	231.39
**W37**								244.15	242.75	261.31	243.55
**W38**	189.47	189.4	189.34	191.88	193.27	195	192.95	195.14	195.1	194.6	194.88
**W39**	194.5	194.25	194.18	193.97	193.94	193.49	194.06	193.89	194.64	195.09	194.59
**W40**	213.85	215.18	215.18	215.35	214.87	214.89	215.25	214.73	216.55	213.55	214.2
**W41**	267.36	267.54	267.93	267.38	267.34						
**W42**								223.56		222.86	223.27
**W43**						230.61		231.25			228.1
**W44**	194.7	194.6	194.5	194.18	194.2	194.18	194.12	194	194.05		194.07
**W45**											
**W46**											

Table S4

Target Well	May_ 2007	Aug_ 2007	Nov_ 2007	Jan_ 2008	May_20 08	Aug_ 2008	Nov_ 2008	Jan_ 2009	May_ 2009	Aug_ 2009	Nov_ 2009
**W01**	276.09	275.92	276.51	276.36	275.11	276.2	276.91	276.11	275.49	277.15	277.26
**W02**	217.79	216.9	217.08	217.02	116.46	215.67	215.75	216.46	214.7	213.91	213.7
**W03**	229.05	227.53	227.98	228.03	226.65	228.11	227.87	226.91	227.4	226.31	226.79
**W04**	240.17	239.16	238.36	239.54	239.14	238.01	237.94	234	239.27	237.44	237.6
**W05**	240.3	237.13	238.66	238.8	238.36	237.92	238.49	240.49	239.49	236.63	239.22
**W06**	209.14	209.15	208.94	208.86	208.42	209.49	209.54	209.42	205.88	206.54	209.19
**W07**	201.45	203.2	202.88	202.34	201.8	202.8	202.62	200.73	201.8	201.7	201.35
**W08**	224.29	227.02	226.32	225.48	225.12	227.77	227.37	226.25	224.34	224.455	224.57
**W09**	258.6	257.33	258.88	257.59	257.6	257.275	256.95	258.21	257.3	255.16	257.43
**W10**	292.69	293.39	293.35	292.975	292.6	292.13	291.7	290.6	293.15	292.7	294.1
**W11**	269.83	270.21	269.48	270.38	269	267.91	271.18	270.5	270.55	270.15	269.65
**W12**	222.2	222.21	222.22	222.23	222.2	221.6	222.24	222.8	221.92	219.78	221.11
**W13**	252.97	251.9	252.65	252.5	252.3	252.43	253.69	254.24	253.28	253.01	253.88
**W14**	218.37	220.14	219.78	219.04	218.05	220.02	219.57	218.94	218.01	219.21	220.08
**W15**	182.5	182.65	182.7	182.47	181.55	181.62	181.52	181.15	183.03	181.92	180.8
**W16**	187.06	186.87	186.9	186.6	186.2	186.4	186.63	186.3	186.22	185.86	185.5
**W17**	203.23	202.83	203.6	203.21	203	203.2	203.6	203.61	203.29	202.81	202.85
**W18**	204.23	204.24	204.17	204.54	203.66	204.41	204.03	204.36	203.28	201.81	203.16
**W19**	191	190.875	190.75	190.55	190.15	189.95	189.98	189.5	189.33	188.81	188.45
**W20**	205.28	203.74	204.57	204.65	204.22	203.08	203.6	203.98	203.85	201.85	202.05
**W21**	213.9	213.65	214.23	214.06	213.31	212.83	214.63	213.88	212.76	210.72	213.28
**W22**	218.92	219.06	218.85	218.95	219.69	220.43	220.36	220.25	219.78	219.32	220.3
**W23**	227.12	225.32	225.15	225.66	225.88	224.46	224.86	225.47	225.47	225.44	221.85
**W24**	217.61	216.14	217.9	217.45	217.01	217.08	217.33	217.52	217.22	216.93	217.25
**W25**	221.7	222.58	222.4	222.1	222.46	222.96	223.45	223.73	222.8	221.93	223.43
**W26**	216.43	215.14	214.78	214.64	215.375	216.11	216.43	216.05	215.67	214.91	215.71
**W27**	303.22	303.72	303.57	302.67	302.42	302.92	303.18	303.07	302.82	302.39	303.73
**W28**	205.69	205.87	206.02	205.7	205.37	206.99	206.52	206.32	206.05	203.04	205.52
**W29**	300.2	301.2	300.58	300.05	299.95	300.72	299.72	300.55	300.03	299.8	301
**W30**	296.28	295.6	296.36	296.26	295.43	295.93	295.98	296.03	295.51	296.185	296.86
**W31**	251.83	251.9	251.9	251.83	252.59	252.63	253.16	253.03	252.06	251.91	252.365
**W32**	225.74	227.04	225.97	224.9	226.7	229.37	228.56	228.46	227.1	223.94	228.39
**W33**	208.75	209.2	208.72	208.7	207.66	209.97	209.62	209.4	208.75	208.05	207.95
**W34**	214.07	213.25	212.43	214.53	214.53	211.68	212.73	213.98	210.59	214.52	212.18
**W35**	207.2	207.39	207.38	206.81	206.61	207.18	207.48	207.21	207.11	206.99	206.23
**W36**	230.8	231.39	231.33	231.34	229.75	229.46	230.68	231.9	230.37	230.65	231.98
**W37**	242.75	241.79	242.02	242.2	242.38		243.02	243.29	242.59		241.5
**W38**	194.87	195.14	195.1	194.85			196.3		195.15	195.16	194.1
**W39**	194.19	194.26	194.39	194.07	192.94	193.24	193.59	193.34	192.98	191.79	191.59
**W40**	214.17	213.04	211.5	213.55	212.47	211.95	212.6	212.94	212.25	211.55	211.4
**W41**	267.24	267.27	267.61	267.59		267.57	267.04	268.49	267.8	267.54	268.32
**W42**	222.61		221.35	221.17	220.65	220.02	220.17	220.64	219.74	218.93	220.32
**W43**	228			226.5	227.25	224.34	224.34	226.89	226.86	220.95	224.13
**W44**	194.04	194.17	194.45	193.95	193.95	193.4	193.93	193.85	193.72	193.7	193.7
**W45**			215.21		216.1	214.28	214.87	215.93	216.88	213.95	213.97
**W46**					223.08	222.5	224.12	224.21	223.54	222.21	221.78

Table S4

Target Well	Jan_ 2010	May_ 2010	Aug_ 2010	Nov_ 2010	Jan_ 2011	May_ 2011	Aug_ 2011	Nov_ 2011	Jan_ 2012	May_ 2012	Aug_ 2012
W01	278.02	275.35	277.71	278.2	278.31	278.66	280.09	278.1		277.06	
W02	214.5	213.88	213.67	213.46	213.96	213.14	212.87	212.68	213.11		
W03	227.2	226.55	226.34	226.14	227.88	226.58	225.85	225.64		226.07	
W04	238.2	238.37	236.65	237.24	238.93	238.93	238.1	236.89			
W05	238.75	238.61	237.07	238.91	239.12	235.8	236.35	237.4			
W06	207.09	206.46	208.5	208.75	208.57	208.81	209.01	208.92		209.08	208.13
W07	201.87	201.99	204.57	205.81	205.44	206.2	205.82	204			
W08	223.94	223.31	224.96	226.55	225.96	224.98	226.36	226.91	225.13	221.87	
W09	257.89		252.44	257.7	258.9	258.1	257.26	258.04			
W10	293.525	292.95	293.98	294.18	293.18	291.2	294.7	294.87			
W11	269.65	269.05	269.59	270.2	270.29	269.72	269.38	269.09		269.65	
W12	220.94	220.7	220.5	220.92	221.2	220.91	220.6	220.5			
W13	253.76		254.22	254.34	254.35	250.6	255.05	255.05			
W14	219.45	218.92	215.95	218.01	220.07	218.79	219.69	219.95	218.5	218.05	219.22
W15	180.6	181.98	182.78	181.3	180.67	180.84	182.4	181.23	179.6	180.71	180.35
W16	185.77	184.97	184.93	185.06	185.09	184.98	184.87	185.4	185.17	184.56	185
W17	203.05	203.3	202.55	203.07	203.39	202.74	203.46	202.95	203.7	203.02	203.01
W18	203.18	202.56	202.01	202.16	201.36	200.96	201.56	201.71	202.24	201.72	198.94
W19	188.55	187.81	187.77	187.9	189.84	187.32	187.48	187.08			
W20	202.74	201.96		202.3	202.99	201.48	199.97	202.3			
W21	212.93	212.11	211.64	214.02	213.67	212.77	212.88	214.5	212.7	212.83	211.43
W22	220.13	218.83	219.43	219.83	218.95	218.68	220.45	220.17	220.88	220.11	219.47
W23	221.85	221.85	222.84	223.33	223.82	224.38	223.94	223.51			
W24	217.47	216.78	216.4	216.72	216.87	216.34	216.505	216.67	216.93	216.47	216.55
W25	223.25	223.38	223.44	223.5	223.7	222.72	223.57	223.6			
W26	215.815	215.92	216.025	216.13	215.92	215.21	215.31	215.35			
W27	303.22	302.32	303.77	304.56	304.3	303.25	304.75	304.2	303.65	302.9	302.3
W28	204.37	203.85	204.4	203.53	204.07	205.49	204.78	204.67		204.67	
W29	300.85	299.95	300.65	301.35	300.28	299.97	302.08	301.89	300.33	299.61	300.24
W30	296.93	295.53	296.78	298.75	298.38	296.91	297.78	297.47			
W31	252.82	251.84	254.94	253.25	253.16	252.46	252.74	253.56			
W32	228.11	223.97	227.84	228.74	228.89	227.465	226.04	227.92		225.52	
W33	207.85	206.37	208.9	210.18	211.91	209.62	209.68	211.68	212.05	211.65	210.9
W34	214.2	211.63	212.93	213.18	215.5	214.3	212.02	212.6	210.9	212.03	211.38
W35	206.13	206.3	206.14	206.58	206.3	206.6	206.31	206.58	206.17	206.4	
W36	231.42	230.23	232.3	232.35	231.44	230.1	231.65	232.12	230.2	230.22	231.5
W37	241.75	241.1	240.95	241.54	241.94	241.3	241.42	242.09	242.36	241.58	240.56
W38		195.33								194.57	
W39	191.29	191.09	192.65	193.99		192.91	192.52	192.39		181.88	
W40	211.6	211.27	211	210.85	211.25	211.25	210.2	209.7	211.01	210.75	208.98
W41	268.41	267.37	267.6	267.74	267.93	267.44	267.54	268.99	268.29	267.84	269.13
W42	220.05	218.47	218.35	218.97	219.9	218.25	218.15	217.92	218.79	217.42	217.64
W43	225.83				226.38			224.58	224.54	225.6	224.22
W44	193.6	193.24	193.29	193.42	193.52	193.48	193.44	199.02	199.06	193.58	
W45	214.63	214.62	214.08	213.49	213.7	214.9					
W46	221.85	221.37	220.63	221.3	221.91						

Table S4

Target Well	Nov_ 2012	Jan_ 2013	May_ 2013	Aug_ 2013	Nov_ 2013	Jan_ 2014	May_ 2014	Aug_ 2014	Nov_ 2014	Jan_ 2015	May_ 2015
W01	277.45	276.05	277.06	279.26	277.9	275.8	277.61	280.06	277.54	279.71	
W02											
W03	227.9										
W04							236.22				234.97
W05						236.5	236.38	233.12	233.9	234.28	235.19
W06	209.07	209.13	208.83	209.49	208.39	209.08	209.51		208.3	208.66	209.49
W07							203.31				203.63
W08	223.49		221.48	224.07	223.58		224.53	224.02	224.57	224.1	223.74
W09							257.66		257.79	271	267.3
W10						294.06	293.32	294		294.1	290.4
W11	270.14	270.22	270	270.72	270.88	270.86	269.63	270.47	270.48	271.09	
W12											
W13						254.71	255.16		254.65	254.93	254.78
W14	220.35	217.92	219.27	221.02	220.76		219.5	219.72	220.01		219.26
W15	180.63	182.3	180.32	182.09	179.3	179.75	180.55		179.82		
W16	184.41	184.4	184.14	183.7	184				183.3		183.69
W17	203.25	203.05	202.93	203.6	203.59	203.32	202.75	203.4	202.55		202.94
W18	202.32	201.16	200.8	201.08	201.64	201.92	201.68	200.39	201.17		201.14
W19							186.2		185.43		186.65
W20						201.85	201.55	197.3	199.46	200.51	199.1
W21	212.43	212.43	211.96	211.73	212.43	212.13	212.08	210.13	211.09	211.38	210.03
W22	219.6	219.74	219.5	220.5	220.6	217.45					
W23											
W24	215.75	215.49	215.69	215.17		216.84	215.55			214.89	214.19
W25											
W26											
W27	302.02	302.34	302.32	304.7	304.17	303.9	303.52	303.9	303.92		303.4
W28	205.42	205.07	205.24		205.35						
W29	301.8	300.25	298.97	300.03	302.82				305.32		301.22
W30					295.87		297.24		297.54		
W31						249.13	251.18		250.92	251.55	255.4
W32	227.95		227.24		226.64		225.14		225.82		224.27
W33	212.14	209	211.29	211.7	212.27		212.57	211.54			212.61
W34	211.11		211.5	211	211.52	210.18	211.03			210.08	210.78
W35	206.8		206.96		208.3	207.88	208.3		207.75		207.73
W36	231.8		230.25	231.3	231.4	231	230.45	229.7	230.55	230.6	229.12
W37	240.4	240.33	240.25	238.84	237.3	239.6	239.8				
W38	195.78										
W39	191.15		186.89	190.49	189.57		189.89		190.11		189.29
W40	207.37	209.55					208.41				
W41	267.85	267.96		268.31	269.38						
W42	216.37	216.57	216.21	218.54	215.2						213.06
W43	221.22	222.88	226.22	225.78	221.01	221.99				221.34	
W44	193.69	193.67	193.52	193.83	194	193.98	193.97	194.09	194.1	194.12	194.1
W45	214.85	214.7	214.56			211.51		213.78		212.72	212.64
W46											

Table S4

Target Well	Aug_ 2015	Nov_ 2015	Jan_ 2016	May_ 2016	Aug_ 2016	Nov_ 2016	Jan_ 2017	May_ 2017	Aug_ 2017
W01				278.26	280.16	277.76	277.07	276.78	
W02									
W03									
W04		233.15		234.61			231.62	232.97	
W05		233.3		235.06	228.97	230.69		233.18	230.57
W06		209.79		208.97		210.26		210.27	
W07		204.05		203.4		204.6		202.7	
W08		224.37		223.17	225.67	225.07	225.66	224.02	227.53
W09	270.42	271.06		256.06		256.4		255.85	
W10	293.52	294.16		294	293.4	293.7	293.3	292.55	292.89
W11		270.14		270.38	268.6	268.42	268.38	267.75	
W12									
W13	254.98			254.71	255.16	254.89	254.45	254.4	254.7
W14		220.9		218.29		219.97		216.49	
W15				183.33	183.7	184.01		185.69	185.4
W16		185.42		182.34		181.61	182.9		
W17		202.28		200.57		201.29		200.45	
W18	200.03	199.1		200.24	197.86	199.39	199.82	199.36	
W19		185.28		185.15		184.48	185.15	184.2	
W20		197.59		197.93		197.25	198.58	197.14	197.45
W21	210.03	210.18		209.29		209.02	209.63		
W22				220.64		221.41		218.69	
W23									
W24		214.01		213.55	214.55	213.07		212.71	209.36
W25									
W26									
W27		304.12		303.02	303.87	303.44		301.12	
W28									
W29									
W30				295.3		294.33			
W31	254.1	251.56	251.62	250.45	250.86	250.4		250.16	250.09
W32									
W33		213.1		212.8	213.6	213.5	213.15	211.79	213.9
W34	210.68	210.92		209.88	210.28	210.28	210.5	210.26	
W35		207.63		208.28		208.03			
W36		229.53		230.13	229.45	229.49	230.35	226.95	230.15
W37									
W38							194.45		
W39		189.66		190.29	189.05	189.94	193.21	186.59	
W40									
W41									
W42	213.06								
W43									
W44	194.43	194.29			194.47	194.55	194.03	194.36	194.31
W45		212.4				210.55	210.65		
W46									

Table S5: Aquifer parameters of the steady-state model.

Aquifer Parameters	Values
K_x (Hydraulic conductivity along X axis)	72 m/day
K_y (Hydraulic conductivity along Y axis)	72 m/day
K_z (Hydraulic conductivity along Z axis)	7 m/day

References

1. Central Ground Water Board (CGWB). *Dynamic groundwater resources of India (As on March 2004)*. (Ministry of Water Resources, Government of India 2006).
2. Central Ground Water Board (CGWB). *Dynamic groundwater resources of India (As on March 2009)*. (Ministry of Water Resources, Government of India 2011).
3. Central Ground Water Board (CGWB). *Dynamic Groundwater Resources of India (As on March 2011)*, (Ministry of Water Resources, River Development & Ganga Rejuvenation, Government of India 2014).
4. Central Ground Water Board (CGWB). *Dynamic Groundwater Resources of India (As on March 2013)*, (Ministry of Water Resources, River Development & Ganga Rejuvenation, Government of India 2017).
5. Chatterjee R, et al. Dynamic groundwater resources of National Capital Territory, Delhi: assessment, development and management Options. *Environ Earth Sci. J*. **59**, 669-686 (2009).
